# The effect of modulated electro-hyperthermia on local disease control in HIV-positive and -negative cervical cancer women in South Africa: Early results from a phase III randomised controlled trial

**DOI:** 10.1371/journal.pone.0217894

**Published:** 2019-06-19

**Authors:** Carrie Anne Minnaar, Jeffrey Allan Kotzen, Olusegun Akinwale Ayeni, Thanushree Naidoo, Mariza Tunmer, Vinay Sharma, Mboyo-Di-Tamba Vangu, Ans Baeyens

**Affiliations:** 1 Department of Radiation Sciences, Radiobiology, University of the Witwatersrand, Johannesburg, South Africa; 2 Department of Radiation Oncology, Wits Donald Gordon Medical Centre, Johannesburg, South Africa; 3 Department of Nuclear Medicine, Charlotte Maxeke Johannesburg Academic Hospital, Johannesburg, South Africa; 4 Department of Radiation Sciences, Radiation Oncology, University of the Witwatersrand, Johannesburg, South Africa; 5 Department of Radiation Sciences, Nuclear Medicine, University of the Witwatersrand, Johannesburg, South Africa; 6 Department of Human Structure and Repair, Radiobiology, Ghent University, Ghent, Belgium; MD Anderson Cancer Center, UNITED STATES

## Abstract

**Background:**

The global burden of cervical cancer remains high with the highest morbidity and mortality rates reported in developing countries. Hyperthermia as a chemo- and radiosensitiser has shown to improve treatment outcomes. This is an analysis of the local control results at six months post-treatment of patients enrolled in an ongoing study investigating the effects of the addition of modulated electro-hyperthermia (mEHT) to chemoradiotherapy for the treatment of HIV-positive and -negative cervical cancer patients in a low-resource setting.

**Methods:**

This ongoing Phase III randomised controlled trial, conducted at a state hospital in Johannesburg, South Africa, was registered with the appropriate ethics committee. After signing an informed consent, participants with FIGO stages IIB to IIIB squamous cell carcinoma of the cervix were randomised to receive chemoradiotherapy with/without mEHT using a secure online random-sampling tool (stratum: HIV status) accounting for age and stage. Reporting physicians were blind to treatment allocation. HIV-positive participants on antiretroviral treatment, or with a CD4 count >200cell/μL were included. mEHT was administered 2/weekly immediately before external beam radiation. The primary end point is local disease control (LDC) and secondary endpoints are toxicity; quality of life analysis; and two year survival. We report on six month LDC, including nodes visualised in the radiation field on ^18^F-FDG PET/CT (censored for six month survival), and six month local disease free survival (LDFS) (based on intention to treat). Trial status: Recruitment closed (ClinicalTrials.gov: NCT03332069).

**Results:**

271 participants were recruited between January 2014 and November 2017, of which 210 were randomised for trial and 202 were available for analysis at six months post-treatment (mEHT: n = 101; Control: n = 101). Six month LDFS was higher in the mEHT Group (n = 39[38.6%]), than in the Control Group (n = 20[19.8%]); *p = 0*.*003*). LDC was also higher in the mEHT Group (n = 40[45.5%]) than the Control Group (n = 20[24.1%]); (*p = 0*.*003*).

**Conclusion:**

Our results show that mEHT is effective as a chemo-radiosensitiser for cervical cancer, even in high risk a patients and resource-constrained settings.

## Introduction

The global burden of cervical cancer remains high, with 570 000 new cases and 311 000 deaths estimated to have occurred in 2018. It is the fourth most common cancer, and the cancer with the fourth highest mortality rate, amongst women. The mortality rate from cervical cancer is around 3 cases per 100,000 in developed countries and around 18 per 100,000 in developing countries.[1]

Current standard of care treatment for locally advanced cervical cancer (LACC), which includes International Federation of Gynaecology and Obstetrics (FIGO) stages IB2 to IVA, is a combination of chemotherapy (most commonly platinum-based), and radiotherapy (RT).[2] The treatment of cervical cancer in developing countries is complicated by limited resources, late stage of disease at presentation,[3] and the high prevalence of Human Immunodeficiency Virus (HIV) and Acquired Immune Deficiency Syndrome (AIDS). Out of 54 African countries, 28 do not have access to radiotherapy treatments. Of those with access to RT, only 20 have access to brachytherapy (BT).[4] Where RT is available, the planning and delivery techniques are often sub-optimal, with many countries still relying on low energy machines, such as Cobalt60. There are usually not enough devices or staff to handle the high patient load, resulting in long waiting times for treatment.[5] The lack of radiology support restricts the availability of sophisticated planning techniques which require computed tomography (CT) or magnetic resonance imaging (MRI). Low-resource settings therefore rely on less labour-intensive 2D planning techniques that do not require sophisticated imaging to manage the burden of the high patient volumes. In our facility patients do have access to BT, however imaging techniques are not routinely available for the planning of oncology treatments. Treatment outcomes in low-to-middle-income countries (LMICs) are significantly worse than in less resource-constrained settings with an estimated 87% of cervical cancer-related deaths occurring in LMICs in 2018.[6] There is a scarcity of information on the outcomes and on the confounding prognostic factors unique to LMICs.[7] The protocols and outcome measures used are often sub-optimal in resource-constrained settings and therefore not comparable to international literature. For example in Uganda, 316 cervical cancer patients were evaluated and authors reported that HIV-positive patients (42%) had more than a two-fold higher risk of death than HIV-negative patients. More than two-thirds of the patients that died in the first year, died in the first 6 months post-treatment and only 32% of the HIV-negative and 35% of the HIV-positive patients were alive at one year post-treatment. While this study did not report on local control, it does highlight the high mortality rates seen in low-resource settings.[8] HIV infection is associated with a higher risk of treatment interruptions and residual disease,[9] a higher risk of not completing the prescribed chemotherapy protocol,[10] and worse survival rates for cervical cancer patients.[11] The high burden of cervical cancer on already strained public health systems in Sub-Saharan Africa necessitates the investigation of feasible chemo-radiosensitisers that are effective, even under such difficult conditions.

Hyperthermia (HT) is well known for its radiosensitising effects and the benefits of its addition to RT for the management of LACC have been described in two meta-analyses.[12,13] Hyperthermia (HT) has been investigated as a chemo- or radiosensitiser in numerous studies on cervical cancer.[14–17] There is evidence that HT may be particularly effective in the treatment of Human Papilloma Virus (HPV) positive cancers as it promotes the degradation of oncogenic proteins.[18] HT as a radiosensitiser has shown to improve survival and local disease control (LDC) in LACC patients,[19] however at least one study has reported worse toxicity, with no improvement in local disease control.[20] As a chemo-sensitiser HT has demonstrated improved outcomes and acceptable toxicity combined with platinum-based cytotoxic agents in the management of residual or recurrent cervical cancer.[14] Phase I/II studies have shown that chemoradiotherapy plus HT for the management of LACC is tolerated well, with no significant increase in treatment related toxicity resulting from the addition of HT.[21,22] Two Phase III trials were published after recruitment for this study began. One was closed early due to slow recruitment.[23] Neither of the trials reported an increase in toxicity, however both trials reported no significant benefit to the addition of HT to chemoradiotherapy. One study used radiative heating,[23] and one study used a capacitive heating device with a power output of 800-1500W.[24] however two phase III trials published after recruitment for this study began, reported no benefit to the addition of HT to chemoradiotherapy.[23,24]

Radiative hyperthermia utilizes external antennas to induce an electromagnetic field using 70 to 120MHz which is coupled into the patient using a water bolus. Complex MRI guided planning systems and thermometry systems have been developed to support these treatments, minimize unwanted hot spot formation, and focus the energy on the tumour. Capacitive heating devices utilize two electrodes to produce an electro-magnetic field by capacitive coupling with the human body, resulting in heating. Both techniques are widely used clinically.[25] Challenges with radiative heating include the higher costs, complexity and sophisticated setting required to administer the treatments. Capacitive heating devices in comparison are more affordable[25] and carry less risk of hot spot formation. Capacitive heating devices frequently make use of intra-tumoral thermometers or locally applied temperature sensors to confirm temperatures, although the invasiveness of the procedures carries certain risks. Challenges associated with capacitive heating involve the effective heating of deep tumours through thick layers of adipose tissue (>2cm).

Hyperthermia faces challenges with acceptance and consistency in results. Szasz *et al* proposed a HT technique in which 13.56MHz capacitive-coupled modulated radiofrequency is applied to selectively target tumour cells based on the differences in the conductivity and permittivity of malignant tissue compared to healthy tissue.[26,27] This selective technique, known as modulated electro-hyperthermia (mEHT), combines the effects of heating and of the electric field to sensitise tumours to treatments.[28] The tumour, having different biophysical properties to healthy tissue, stores a charge thereby heating up selectively. Once the maximum energy has been absorbed, the excess energy passes through the tumour. This can also be described as extracellular heating where the extracellular matrix is heated with minimal impact on the intracellular environment. The device therefore uses the energy (measured in Kilojoules) that is put into the system, rather than the temperature, as the measured dose. The energy output from the first electrode is compared to the energy received by the second electrode. Using algorithms the absorbed energy can be determined[29–31] and the tumour temperature is calculated. This technique therefore does not require intratumoural or MRI thermo-monitoring systems due to the real-time calculation of temperature by the device based on the energy dose. Confirmation of the heating capacity of the mEHT has been demonstrated.[32,33] The techniques is also able to safely heat tumors, regardless of tumour depth or adipose layer thickness.[30] Hyperthermia interferes with protein synthesis,[26,34] and inhibits DNA and RNA synthesis and repair[34,35] by inducing protein aggregation in the nucleus and changes in the DNA repair foci which inhibit the DNA double-strand break repair processes. This is synergistic for RT and for certain cytotoxic agents which cause DNA double-strand breaks.[36] Hypoxia is a central causative factor for radio-resistance.[37] Heating the tumour also results in changes in blood perfusion and re-oxygenation. [38] Enhanced perfusion results in improved oxygenation in the target tissue, which enhances the effects of the Reactive Oxygen Species induced by IR.[39] Increased temperatures result in an increased immune response at the heated site.[27,40] HT has direct actions on the immune system through direct heat-mediated cell killing, and indirect actions by modulating the innate and adaptive immune system.[41] The extra-cellular heating method used by mEHT minimises the stress on the intracellular environment, reducing the risk of thermo-tolerance. Heating up the extracellular matrix with low frequency mEHT has only a minor impact on the intracellular environment and the primary action and heating occurs in the extracellular matrix and at the cellular membrane.[42] According to research conducted by Andocs *et al*, cell stress induced by mEHT results in the generation of DAMP signals and the application of mEHT could potentially induce local immunogenic cell death.[43] mEHT has also been shown to induce apoptosis [44] more readily than HT.[45] Andocs *et al* hypothesise that the energy absorption at the cell membrane results in a non-homogenous energy distribution causing damage which activates the cell death-related signalling pathways during mEHT.[45]

mEHT has shown benefits and safety as a chemo-sensitiser for the treatment of recurrent or residual cervical cancer[17] and the increase in temperature achieved in cervical tumours using mEHT is similar to the increased temperature reported using other HT methods.[46] Locations in which the technique has shown safety include the brain,[47] head and neck,[48] lung,[49] and pancreas,[50] The different dose monitoring system and temperature calculations means that MRI monitoring is not required. This technique is simple to use in comparison to other technologies, making it suitable for investigating as a chemo-radiosensitiser in a low-resource setting.

In South Africa, cervical cancer is the leading cause of cancer death in women with a lifetime risk of 1 in 44 of developing cervical cancer.[51] According to UNAIDS, in 2017 there were 36.9 million people infected with the HIV world-wide with an estimated 7.1 million of those people living in South Africa.[52] The risk of cervical cancer is significantly increased in HIV-positive patients, likely a result of the of HIV-associated immune suppression[53] and the subsequent increased risk of chronic HPV infection.[54] This, combined with constrained resources, makes South Africa a particularly challenging setting in which to test chemo-radiosensitisers.

We report on an analysis of the local control results at six months post-treatment of patients enrolled in an ongoing study investigating the effects of the addition of modulated electro-hyperthermia (mEHT) to chemoradiotherapy for the treatment of LACC patients in a low-resource setting, with specific interest in the effects on the HIV-positive subgroup. Six month local disease-free-survival (LDFS) is also reported in order to account for the participants who did not survive six months post-treatment. To our knowledge, this is the first phase III randomised controlled trial (RCT) on the trimodality treatment of cervical cancer using this form of HT, the first trial to apply HT twice a week for LACC, the first HT trial in Africa, and the first phase III HT study to include HIV-positive patients in a low-resource setting.

## Materials and methods

This a single centre, phase III, randomised controlled (stratum: HIV status; accounting for stage and age) parallel group trial being conducted at the Charlotte Maxeke Johannesburg Academic Hospital (CMJAH), a public hospital in Gauteng, South Africa, by the Radiation Sciences department of the University of the Witwatersrand. Approval from the Human Research Ethics Committee was obtained (M1704133) on 5^th^ June 2012. According to the requirements of the local ethics board, the trial was registered on the South African National Clinical Trials Register before recruitment was started (ID: 3012). Registration of the trial ClinicalTrials.gov (NCT03332069) was finalised after recruitment began. The authors confirm that all ongoing and related trials for the intervention are registered on ClinicalTrials.gov.

### Eligibility

Patients were recruited by the multi-disciplinary team of gynaecology and oncology specialists at the CMJAH (January 2014 to December 2017) after undergoing clinical staging according to FIGO staging criteria,[55] by chest x-ray, ultrasound and physical assessment. Written informed consent was obtained and participants were referred for additional haematological investigations (liver function; urea and creatinine levels; full blood count; pregnancy test with serum βHCG; HIV test; CD4 count if indicated), and a Positron Emission Tomography/Computed Tomography (PET/CT) scan with ^18^F-Fluorodeoxyglucose (FDG), as part of the screening process.

#### Eligibility criteria

Primary, treatment-naïve, histologically confirmed invasive squamous or adeno-squamous cell carcinoma of the cervix; FIGO stages IIB (with invasion of the distal half of the parametrium), to IIIB (clinically staged); Eligible for CRT with radical intent; >18years old; Eastern Cooperative Oncology Group (ECOG) score <2; Creatinine clearance >60mL/min; Haemoglobin, platelet count and absolute neutrophil count within normal ranges; HIV positive patients on antiretroviral therapy or with a CD4 count >200cell/μL; Ability to understand and the willingness to sign a written informed consent document; Life expectancy >12 months; Patients who underwent emergency RT in the form of brachytherapy for haemostasis prior to enrolment were screened and enrolled provided they met all other eligibility criteria; Body Mass Index (BMI) within normal ranges; Life expectancy of greater than 12 months; Participants with para-aortic and other nodes outside of the radiation field visualised on the ^18^F-FDG PET/CT scans were included.

#### Exclusion criteria

Bilateral hydronephrosis; Second primary malignancy; Extra-pelvic visceral disease visualised on the ^18^F-FDG PET/CT; Vesicovaginal fistula or vesicorectal fistula that required a change in treatment protocols; Abnormal liver function tests; Currently pregnant or breast feeding; Prior hysterectomy; Prior malignancy treated in the preceding two years; Cardiovascular disease (excluding controlled hypertension); Acute or life-threatening infections or medical conditions or life threatening AIDS-defining illnesses other than cervical cancer; Evidence of resistance to antiretroviral therapy in HIV-positive patients; A medical or psychiatric illness that preventing the participant from being able to sign an informed consent or affecting the participant's ability to comply with the protocol stipulations; Carcinoma of the cervical stump; Contra-indications to mEHT (pacemakers, large metallic implants in the treatment area, inability to feel temperature in the treatment area, inability to express or vocalise pain); Any other contraindications to any of the prescribed treatments. Visualisation of para-aortic lymph nodes on planning CT was also an exclusion criterion, however patients were not 3D planned.

### Randomisation and masking

Once participants passed all screening evaluations, participant data was collected and captured by the research co-ordinator using REDCap (Research Electronic Data Capture), an online, secure web based application hosted by the University of the Witwatersrand. The REDCap stratified on-line software generated random-sampling tool was used to randomise participants to receive either CRT alone (Control Group) or combined with mEHT (mEHT Group); stratum: HIV status. Randomisation accounted for age (<30 years; 30–50 years; >50 years), and FIGO stage (IIB; IIIA; IIIB). Participants were informed in a private consultation by the research co-ordinator of their treatment group at treatment verification. The research co-coordinator was not involved in the treatment or clinical evaluations. Double blinding of the participants was not possible due to logistical challenges such as creating a “placebo treatment” on the mEHT device in which the participant would feel heat on the surface but not actually be treated. Clinicians examining the participants during follow-ups were blind to treatment allocation. Physicians reporting on the ^18^F-FDG PET/CT images were blind to treatment allocation and did not interact with the participants, eliminating the risk of biased reporting.

### Treatment

#### Chemotherapy (CHT)

As per the institutional protocol, two doses of cisplatin (80mg/m^2^) administered 21 days apart were planned during external beam radiation (EBRT), subject to the patient’s ability to receive cisplatin (creatinine clearance >60mL/min; acceptable full blood count results; acceptable ECOG status). All participants who were treated with cisplatin received prehydration with normal saline and electrolytes as well as prophylactic antiemetics intravenously, as per the institutional protocol.

#### Radiotherapy

A haemoglobin (Hb) level above 10g/dL was required for EBRT and participants were transfused as needed. All participants were prescribed RT in the form of EBRT (50Gy in 25 fractions of 2Gy), and High Dose Rate (HDR) brachytherapy (BT) (3 fractions of 8Gy). The EMBRACE II study aims for an EQD2 dose of >85Gy. However this is not easily achievable without image guided brachytherapy.[56] The European Society for Medical Oncology Guidelines on Cervical Cancer management lists the optimal treatment as 80–90Gy at point A.[2] We planned an intended total equivalent RT dose of 86Gy. Standard planning for EBRT in our facility is 2D planning, and not the more labour-intensive planning techniques that are standard of care internationally. This is due to the limited staff capacity and resources available to treat the high volume of gynaecological oncology patients seen each year and due to the lack of access to imaging techniques, such as MRIs, for planning purposes. Participants for this study were planned using virtual simulation and standard fields were placed but volumes were not contoured as is standard of care for cervical cancer. EBRT was planned using CT guidance and volumes were determined using bony landmarks to encompass the entire pelvis including 2cm margins from the pelvic side wall laterally. The superior border was the midline of L5 and the inferior border extended to 2cm below the tumour. Posterior border encompasses the sacrum to the third sacral vertebra and the anterior border extended to the inferior border of the pubic symphysis. A four field box techniques was used for planning and the dose prescribed was normalized to 95%. The dose to the nodes was the same as the dose to the centre as it was a homogenous dose distribution to the entire field. 15MV or 18MV photon energy linear accelerators (Siemens Pty, Ltd) were used for the four field technique. ^18^F-FDG PET/CT images were additions to the standard protocols with the sole purpose of monitoring disease response and the costs of the imaging studies were covered by a research grant. The ^18^F-FDG PET/CT images were not used to plan treatments. Para-aortic nodes seen on PET/CT images were therefore not targeted with EBRT. While extended fields to include para-aortic nodes significantly decreases the risk of distant metastases and of para-aortic failure, the effects on overall survival are not yet confirmed[57] and extended fields are not standard in a resource-constrained setting such as ours.

HDR BT was given as per the institutional protocol. Patients received 36Gy equivalent (EQD2 –equivalent dose in 2Gy fractions for an alpha-beta ratio of 10), to point A by intracavity BT using standard tandem-ring or tandem-ovoid vaginal applicators (source used: Iridium 192). 2-dimentional calculations were done based on orthogonal x-rays. Chemotherapy was not permitted on the days that BT was given. A 2D technique was used for the brachytherapy, due to lack of availability of imaging techniques. If the tumour was confined to the upper vagina, a ring-tandem applicator was used. Where the tumour extended inferiorly below the upper half of the vagina, a Joss-Flynn ovoid-tandem applicator was used to cover the whole extent of the vagina. The lower part of the vagina was kept within rectal tolerance when treating with a Joss-Flynn ovoid-tandem applicator. Standard IRCU38 bladder and rectal dose points were calculated for BT. More sophisticated BT techniques require sophisticated imaging techniques which are not available in low-resource settings. The first BT was planned to be administered after 10–13 fractions of EBRT with a week between each subsequent BT dose with the aim of completing all radiation treatments within 52 days.

#### Hyperthermia

Hyperthermia was delivered via a capacitive heating device (Model: EHY2000+; Manufacturer: Oncotherm GmbH, Troisdorf, Germany) which uses 13.56MHz modulated radiofrequency energy. The power was applied using two electrodes, one fixed in the bed and one in an adjustable arm placed over the treatment area. [58] A 30cm diameter applicator with electrode was used to cover as much of the pelvic field as possible. Participants randomised into the mEHT Group received two mEHT treatments (per week at 42.5ºC for a minimum of 55 minutes, plus the prescribed CRT. A step-up heating protocol was used starting at a power output of 60W and increasing to the planned power over a period of five minutes. The planned power for the first treatment was 90W, for the second treatment the planned power was 110W, and the third and subsequent treatments were planned for 130W. An average energy dose of 360KJs over ten treatments was planned. mEHT treatments were administered at least 48 hours apart to reduce the risk of developing thermo-tolerance and EBRT was administered within 30 minutes of completing mEHT. mEHT was not administered on the same day as BT or cisplatin.

#### Follow-up

Participants were examined, and adverse events were documented, weekly on treatment and at six weeks, three months and six months post-treatment. Regular full blood count, renal, and liver function tests were performed. Toxicity was graded according the Common Toxicity Criteria for Adverse Events (CTCAE) version 4 and toxicities worse than Grade 2 were reported to the investigators. The six month follow-up period extended from February 2014 to August 2018 with the final participant completing treatment in January 2018.

### Outcome measures

The primary outcome is LDC at six months post-treatment. Secondary outcomes reported in this paper include disease pattern analysis, early toxicity, and quality of life. As the participants were treated with EBRT to the whole pelvis and HT to the pelvis, we considered the entire region that was within the treatment field, assessing local disease control and not only tumour response. LDC was therefore considered a failure if the presence of disease in the radiation field was noted on any one of the evaluation methods post-treatment (Persistent Disease). The same method of evaluation is described in the protocols of the multicentre (including our centre) study by the AIDS Malignancy Consortium (AMC) evaluating 2D planned EBRT, HDR BT and cisplatin in patients receiving ART (ClinicaTrials.Gov ID:NCT01590017).[59] The results on LDC from this trial are not yet available. Six month LDFS is also reported, taking into account death as a possible risk factor.

All participants were planned to have an ^18^F-FDG PET/CT scan at six months post-treatment to evaluate local disease response and disease patterns presentation using the PERCIST 1.0 criteria (Complete Metabolic Response [CMR] SUV<2.5; Partial Metabolic Response [PMR] >30% reduction in SUV; Stable Metabolic Disease [SMD] <30% reduction in SUV; Progressive Metabolic Disease [PMD] increase in SUV).[60]. 13 participants were unable to have follow-up ^18^F-FDG PET/CT scans due to poor ECOG status or renal dysfunction and were evaluated by Fine Needle Aspiration (FNA) of palpable nodes (n = 1), by CT scan (n = 3), and by clinical examination/PAP smear (n = 9). Evidence of disease on any of the evaluation methods was regarded as treatment failure. Six month LDFS was analysed according to the intention to treat principle and was considered a failure if there was a disease-related death or failure of LDC. We report on the tumour response determined clinically by examination, and on the level of agreement between tumour response determined by clinical exam and as visualised on ^18^F-FDG PET/CT images. The early toxicity and quality of life are reported separately. Further papers on late toxicity and two year survival are planned.

### Statistics

The sample size was calculated based on the estimated required sample sizes for a two-sample comparison of survivors’ functions at two years (statistical power of 90%). We estimated an expected reduction in mortality at two years of 50%, based on survival of 20% in the control group and 40% in the experimental group. The statistical significance is defined as a two-sided alpha<0.05 for a log-rank test, with a constant Hazard ratio of 0.5693. Initially a 15% screening failure rate was predicted, however the screening failure rate was higher than expected (30%) and recruitment continued until the pre-determined required number of 200 eligible participants were enrolled.

Pearson’s Chi squared test and Fisher’s exact tests were used to determine the difference in frequencies between groups. T-test was used to compare the difference in means between groups. Logistic regression was used to test if prognostic factors (treatment arm, number of cisplatin doses; age; overall treatment time; RT dose; CD4 count; Body Mass Index [BMI]; number of mEHT treatments; energy dose administered), significantly predicted outcomes. The three stratification factors were used in the multivariate logistic regression model. Two sided p values are reported and p values <0.05 were considered significant. STATA 13.0 Statistics software program (Stata Corporation, College Station, Texas, USA), was used to analyse the data.

## Results

**Fig 1:**
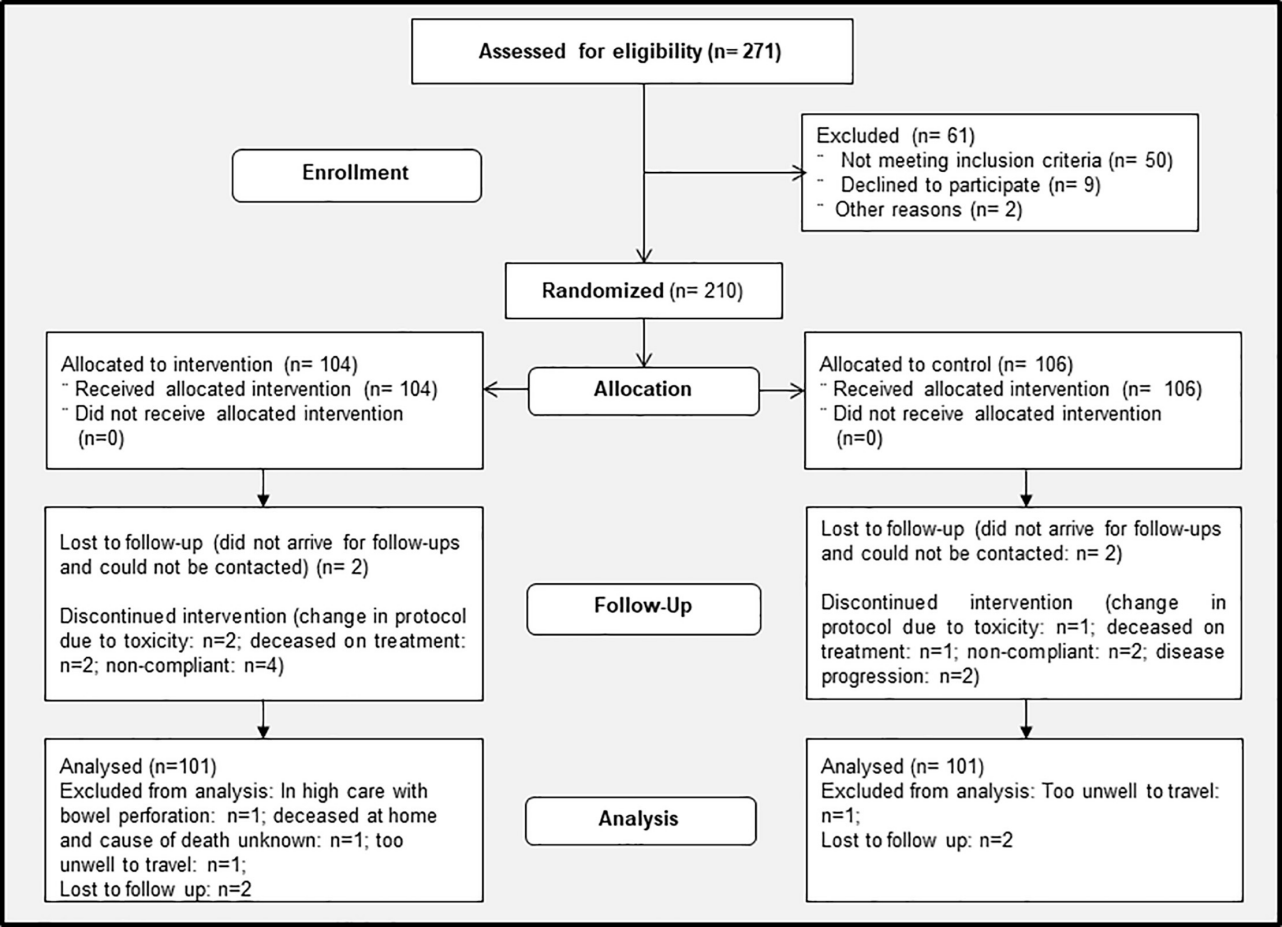
Trial profile: Consort flow diagram. Abbreviations: mEHT: Modulated Electro-Hyperthermia; LDFS: Local Disease Free Survival.

Of 271 screened participants screened between January 2014 and November 2017, 210 were randomised for trial (Fig 1). The most common reasons for omission of the second cycle of cisplatin were renal dysfunction (mEHT: n = 19; Control: n = 11) and delayed in the administration of the first cycle (mEHT: n = 10; Control: n = 7), followed by non-compliance, anaemia, vomiting and dehydration, sepsis, poor ECOG, immunosuppression and other, including supply and technical problems. Reasons for all screening failures can be found in S1 Fig. By the stratification to tumour stage, participant age, and HIV status, the numbers of participants in each group were balanced. Four randomised participants did not arrive for treatment and no further data is available on these participants. They were classified as “lost to follow up”. Patient, tumour and treatment characteristics were balanced in both treatment groups with no significant difference in the means or frequencies (Table 1). There were 104 and 102 participants treated in the mEHT and Control Groups respectively. Four participants could not be evaluated for LDC at six months post-treatment (in high care with bowel perforation: n = 1; deceased at home and cause of death unknown: n = 1; too unwell to travel: n = 2). ^18^FDG-PET/CT scans were conducted at a median of 186 days after completion of all RT treatments (range 154–610 days).

**Table 1:** Participant and treatment characteristics.

		OVERALL	mEHT	Control	P Value
	n	*206*	*104* [50.5%]	*102* [49.5%]	
**HIV Status**	Positive	*105* [51%]	*51* [49%]	*54* [53%]	*p = 575*
	Negative	*101* [49%]	*53* [51%]	*48* [47%]	
**FIGO Staging**	IIB	*75* [36%]	*40* [38%]	*35* [34%]	*p = 0*.*825*
	IIIA	*2* [1%]	*1* [1%]	*1* [1%]	
	IIIB	*129* [63%]	*63* [61%]	*66* [65%]	
**ECOG**	0	*10* [5%]	*3* [3%]	*7* [7%]	*p = 0*.*184*
	1	*196* [95%]	*101* [97%]	*95* [93%]	
**Race**	African	*191* [93%]	*96* [92%]	*95* [93%]	*p = 0*.*035*
	Caucasian	*5* [2%]	*4* [4%]	*1* [1%]	
	Other	*10* [5%]	*4* [4%]	*6* [6%]	
**Age [years]**	Mean	48.6	48.0	49.2	*p = 0*.*388*
	SD	10.49	10.77	10.22	
	Range	26–72	26–68	28–72	
**BMI**	Mean	27.45	27.81	27.08	*p = 0*.*431*
	SD	6.68	6.95	6.41	
	Range	15–49	15–49	15–41.7	
**Hb [g/dL]**	Mean	10.95	10.96	10.94	*P = 0*.*924*
	SD	2.26	2.13	2.39	
	Range	5.2–16.2	5.7–16.2	5.2–15.2	
**CD4 count [cells/μL] in HIV positive**	Mean	550	576.5	524.9	*P = 0*.*315*
	SD	261.76	251.57	270.97	
	Range	95–1524	148–1204	95–1524	

Abbreviations: mEHT: Modulated electro-hyperthermia; HIV: Human Immunodeficiency Virus; FIGO: International Federation of Gynaecology and Obstetrics; ECOG: Eastern Cooperative Oncology Group; SD: Standard Deviation; BMI: Body Mass Index; Hb: Haemoglobin.

### Treatment characteristics

Treatment characteristics are summarised in Table 2. Participants with a decrease in creatinine clearance to <60mL/min, and in whom measures to improve creatinine clearance failed, or whose CD4 count decreased to <150cells/uL, were not prescribed the planned cisplatin. Mean days to complete all radiation was 41.79 days (range 0–97). Six participants in the mEHT Group and four participants in the Control Group received less than 50Gy EBRT. Three participants died during on treatment (mEHT: n = 1; Control: n = 2), two did not arrive for treatment, and two were moved to palliative care due to progression on treatment. Three participants who missed three or less fractions of EBRT cited transport difficulties as the reasons. There were no significant differences in early toxicities between the two groups. The mean dose of cisplatin received was 1.3. The main reasons for not receiving any cisplatin, a delayed first dose of cisplatin, or the cancellation of the second dose of cisplatin, are renal dysfunction (27 participants in the mEHT Group and 22 participants in the Control Group) and anaemia (n = 7 in both treatment groups). Other reasons for not receiving the required dose of cisplatin include leucopoenia; dehydration; sepsis; poor ECOG; and a low CD4 count. Three participants in the mEHT Group had a reduction in power during the mEHT treatments by more than 20% due to reported pain at the treatment site.

**Table 2:** Treatment characteristics.

		OVERALL	mEHT	Control	P Value (t-test)
	n	*206*	*104* [50.5%]	*102* [49.5%]	
**Total radiation dose**	Mean	84.3Gy	84.7Gy	84Gy	*p = 0*.*624*
	SD	9.55	7.65	11.18	
	Range	2-86Gy	20-86Gy	2-86Gy	
**Total external beam radiation**	Mean	48.2Gy	49.4Gy	48.0Gy	*p = 0*.*724*
	SD	8.47	7.74	9.19	
	Range	2-50Gy	20-86Gy	2-50Gy	
**Days to complete all RT**	Mean	41.79	41.46	42.13	*P = 0*.*560*
	SD	8.16	5.88	9.98	
	Range	0–97 days	12–65 days	0–97 days	
**No of Cisplatin doses**	Mean	1.31	1.38	1.25	*p = 0*.*179*
	SD	0.69	0.67	0.58	
	0	*27* [13%]	*11* [11%]	*16* [16%]	
	1	*88* [43%]	*43* [41%]	*45* [44%]	
	2	*91* [44%]	*50* [48%]	*41* [40%]	
	Mean	2.88	2.89	2.87	*p = 0*.*778*
	SD	0.55	0.52	0.58	
**No of brachytherapy fractions**	0	*2* [1%]	*1* [1%]	*1* [1%]	
	1	*2* [1%]	*0* [0%]	*2* [3%]	
	2	*4* [2%]	*3* [4%]	*1* [1%]	
	3	*198* [95%]	*100* [95%]	*98* [95%]	
**No of mEHT doses**	Mean		9.09		
	SD		2.07		
	Range		1–10		
**Average administered (KJ)**	Mean		369.32KJ		
	SD		71.06		
	Range		237- 429KJ		

Abbreviations: mEHT: Modulated electro-hyperthermia; SD: Standard Deviation; RT: Radiotherapy; KJ: Kilojoules.

### Local disease control and local disease free survival

In total, 202 participants were eligible for six month LDFS analysis (mEHT: n = 101; Control: n = 101), of which 171 participants (mEHT: n = 88 [87.1%]; Control: n = 83 [82.2%]), were alive at six months post-treatment. Complete metabolic response (CMR) of all local disease in the radiation field (LDC) in the participants who survived at six months was significantly associated with mEHT administration with a LDC seen in 40[45.5%] of the mEHT Group participants compared to 20[24.1%] of the Control Group (Pearson’s chi2: *p = 0*.*003*). In a multivariate logistic regression model adjusted for HIV status, FIGO stage and age (Table 3), participants in the mEHT Group were significantly more likely to achieve a LDC than participants in the Control Group (OR: 0.39, 95% CI: 0.20–0.77; p = 0.006). Age was also a predictor of LDC (OR: 1.04, 95% CI: 1.00–1.08; p = 0.040).

**Table 3:** Result of Multivariate logistic regression model to predict LDC (adjusted for HIV status, age, and stage).

Variable	Odds Ratio [OR]	P>|z|	95% Confidence interval [CI] range
Arm	0.39	0.006	0.20–0.77
HIV status	0.91	0.781	0.45–1.83
Age (years)	1.04	0.040	1.00–1.08
Stage (II or III)	1.09	0.604	0.78–1.54

Abbreviations: HIV: Human Immunodeficiency Virus

The intention to treat analysis showed a significant association between six month LDFS and the administration of mEHT (six month LDFS in mEHT Group: n = 39 [38.6%]; six month LDFS in Control Group: n = 20 [19.8%]; Pearson’s Chi2: *p = 0*.*003*). A multivariate logistic regression model for six month LDFS (Table 4) also showed that participants in the mEHT Group were significantly more likely to achieve a six month LDFS than participants in the Control Group (OR: 0.36, 95% CI: 0.19–0.69; p = 0.002) and age was also a predictor of six month LDFS (OR: 1.04, 95% CI: 1.00–1.08; p = 0.031).

**Table 4:** Result of logistic regression analysis to predict six month LDFS (adjusted for HIV status, age, and stage).

Variable	Odds Ratio [OR]	P>|z|	95% Confidence interval [CI] range
Arm	0.36	0.002	0.19–0.69
HIV status	0.91	0.788	0.45–1.83
Age (years)	1.04	0.031	1.00–1.08
Stage (II or III)	1.04	0.830	0.75–1.44

When analysed by HIV status, six month LDFS occurred in n = 27 [26.5%] of the HIV positive participants (n = 102), and n = 32 [32%] of the HIV negative participants (n = 100). The association between HIV status and six month LDFS was not significant (Pearson’s Chi2: *p = 0*.*338*). When analysed by HIV status and treatment group, six month LDFS is higher in the HIV-positive and -negative participants treated with mEHT, with the highest six month LDFS seen in the HIV negative mEHT Group. The association between mEHT and six month LDFS was significant in the HIV-negative participants (Pearson’s Chi2 *p = 0*.*006*), but was not significant in the HIV-positive participants (Pearson’s Chi2 *p = 0*.*174*).

### Tumour response

**Fig 2:**
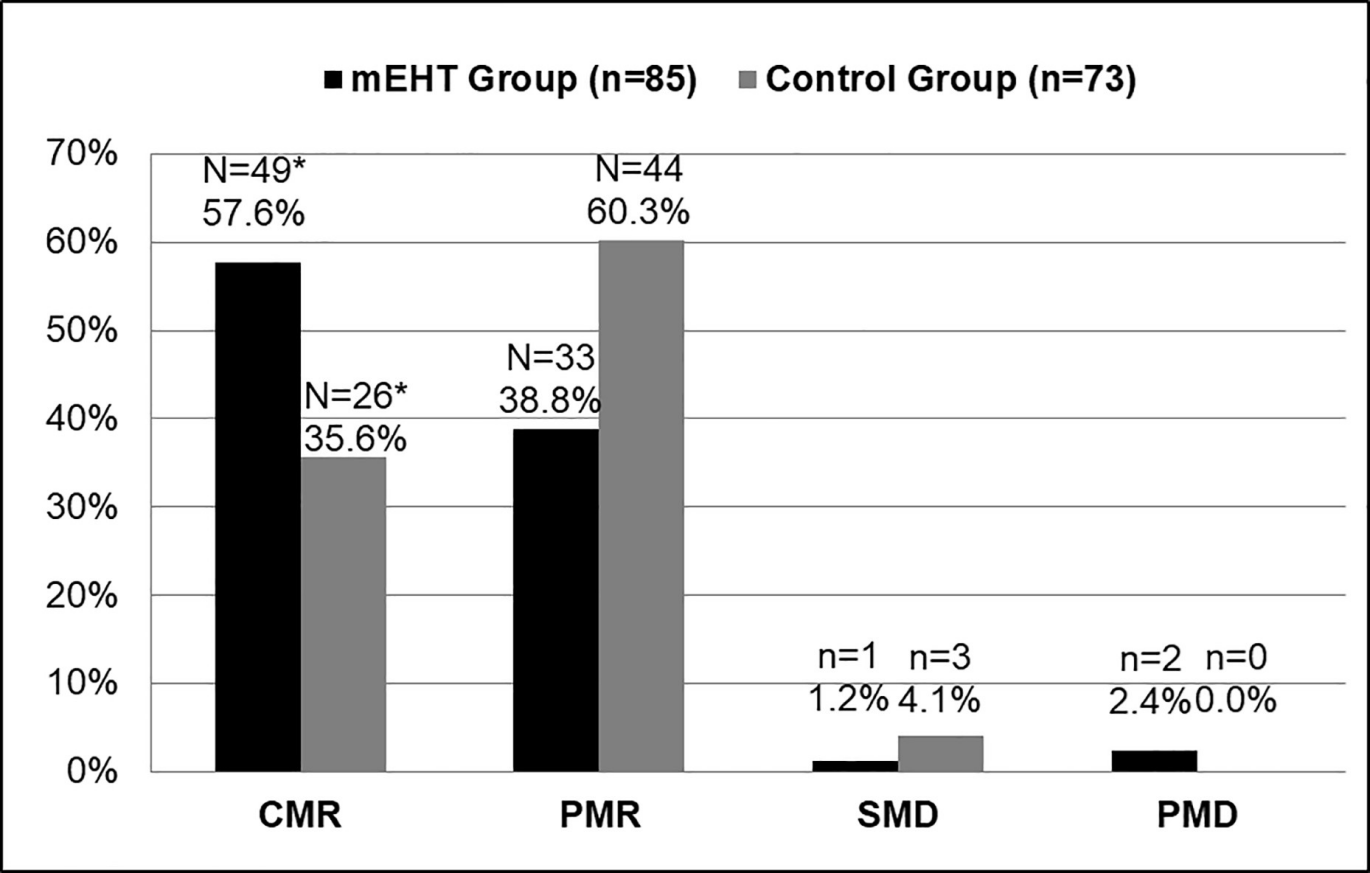
Tumour Response as Seen on ^18^F-FDG PET/CT (PERCIST 1.0) by Treatment Group. mEHT: Fischer’s exact table of association between all four metabolic responses and mEHT: *p = 0*.005*. Abbreviations: mEHT: Modulated electro-hyperthermia; CMR: Complete Metabolic Response; PMR: Partial Metabolic Response; SMD: Stable Metabolic Disease; PMD: Progressed Metabolic Disease.

CMR of the tumour in the 158 participants (mEHT group: n = 85 [HIV-positive: n = 40; HIV-negative: n = 45]; Control Group: n = 73 [HIV-positive: n = 35; HIV-negative: n = 38), who were eligible for ^18^F-FDG PET/CT scans at six months post-treatment was higher in the mEHT Group (n = 49 [57.6%]) than in the Control Group (n = 26 [35.6%]), with a significant association between the metabolic responses and the administration of mEHT (Fischer’s exact: *p = 0*.*005*). These results are represented graphically in Fig 2. Complete metabolic response was higher in HIV positive participants (n = 40 [53.3%]), than the HIV-negative participants (n = 35 [42.2%], but this association was not significant (Fisher’s exact: *p = 0*.*340*).

**Fig 3:**
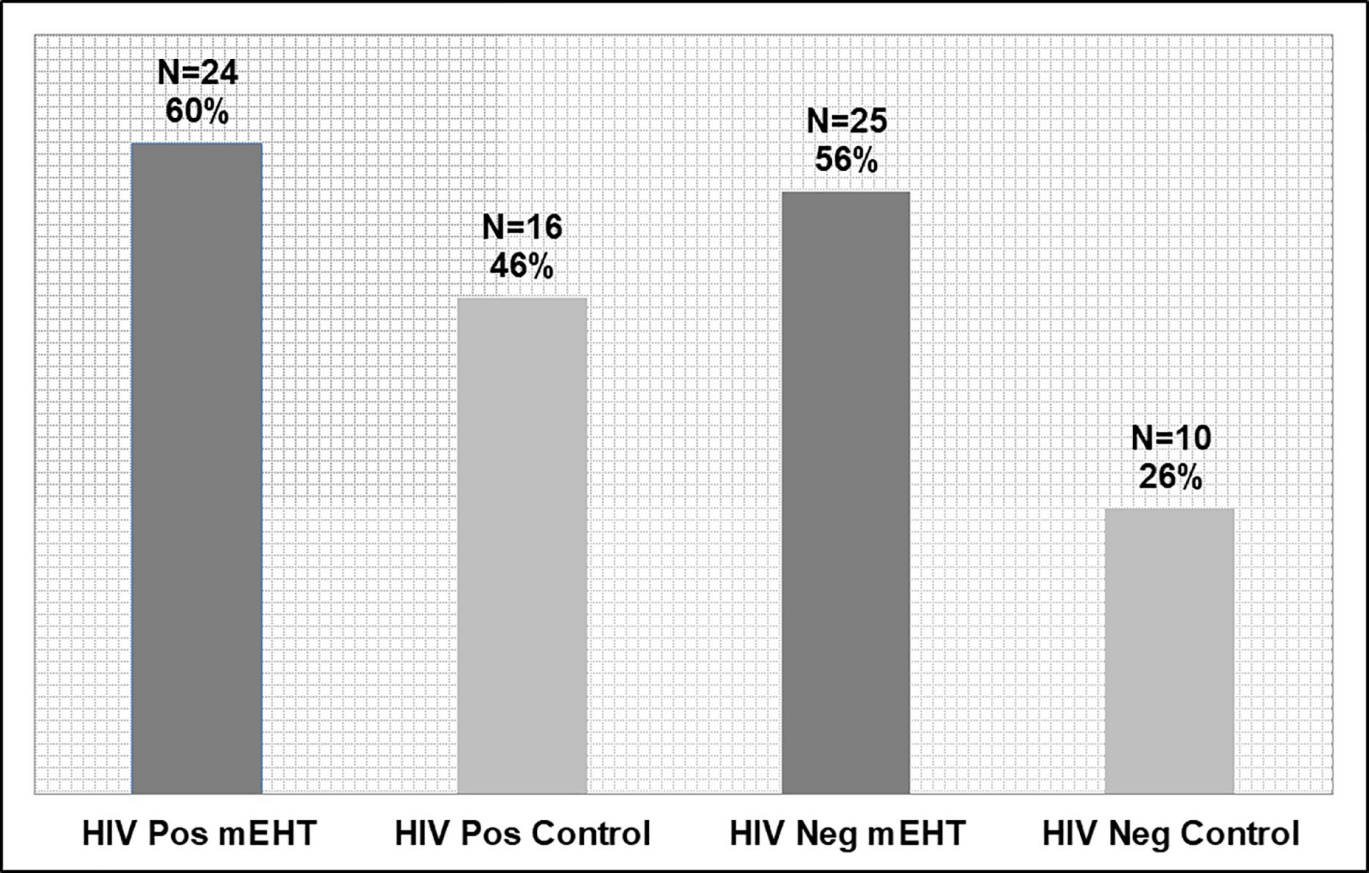
Tumour Response on ^18^F-FDG PET/CT (PERCIST 1.0) by Treatment Group and HIV Status. Total participants in each subgroup: HIV-Positive mEHT: n = 40; HIV-Positive Control: n = 35; HIV-Negative mEHT: n = 45; HIV-Negative Control: n = 38. Abbreviations: mEHT: Modulated electro-hyperthermia.

In an analysis by HIV status and by treatment group, the CMR was higher in the mEHT Group than in the Control Group in both HIV-positive and HIV-negative participants. The association between the CMR rates and mEHT was larger in the HIV-negative participants (mEHT: n = 25 [56.5%]; Control: n = 10 [26.3%]; Fisher’s exact: p = 0.006), than in the HIV-positive participants (mEHT: n = 24 [60.0%], Control: n = 16 [45.7%]; Fisher’s exact: p = 0.196), as seen in Fig 3.

### Prognostic variables

The regression analyses show that age, number of cisplatin doses, treatment time, RT dose, and CD4 count, were not significant predictors of six month LDFS in our study (S1 Table).

In order to assess the efficacy of mEHT further, we considered the effect of BMI[31] (S2 Fig), energy dose (S3 Fig), and number of mEHT treatments on tumour response. Multivariate regression analyses showed that these variables were not a predictor of six month LDFS (S2 Table).

### Disease pattern analysis

In the mEHT Group (n = 95), there were 15[37.5%] participants with extra-pelvic visceral disease visualised on the post-treatment ^18^F-FDG PET/CT scans (HIV positive: 9[23%] out of 40; HIV negative: n = 6[13%] out of 45). The Control Group (n = 73) had 16[45.7%] participants with visualised extra-pelvic visceral disease (HIV positive: 7[20%] out of 35; HIV negative: n = 9[24%] out of 38).The most common site for distant metastasis at six months was lung (n = 20), followed by liver (n = 12), bone (n = 9); soft tissue (n = 1), and brain (n = 1). Ten participants had multiple sites of distant visceral metastases.

54 Participants (64%) from the mEHT Group and 54 participants (74%) from the Control Group had nodes outside of the radiation field visualised on the pre-treatment ^18^F-FDG PET/CT scans. The location of the nodes visualised on the pre-treatment scans are listed in Table 5. Para aortic nodes were visualised on the pre-treatment scans in 26 of the mEHT Group participants (25%) and 29 of the Control Group participants (28%). We compared the pre-treatment ^18^F-FDG PET/CT scans of participants with extra-pelvic nodes to the post-treatment scans of the same participants in order to determine the response, if any, of the disease outside the radiation field. The percentage of participants who showed a systemic response to treatment, with resolution of all metabolically active disease (including extra-pelvic nodes which were not in the RT or mEHT fields, pelvic nodes and the primary tumour), was significantly higher in the mEHT Group: 24.1% (n = 13; HIV-positive: n = 6; HIV-negative: n = 7), versus the Control Group: 5.6% (n = 3; HIV-positive: n = 1; HIV-negative: n = 2) (Chi2, Fisher’s exact: *p = 0*.*007*). One participant in each group did not receive cisplatin. In a multivariate analysis of age, cisplatin doses, total radiation dose and the number of days between the final radiation treatment and the follow-up ^18^F-FDG PET/CT, none of the variables were indicators of an abscopal effect.

**Table 5:** Number of patients with nodes visualized in each region on pre-treatment scans.

	mEHT (n = 54)		Control (n = 54)	
Head and Neck	30	[56%]	26	[48%]
Thorax	29	[54%]	30	[56%]
Abdomen	32	[59%]	39	[72%]
Pelvis	43	[80%]	46	[85%]

Abbreviations: mEHT: Modulated Electro-Hyperthermia

### Toxicity

In our study, mEHT did not have any effect on the frequency of CRT-related early toxicities. The administration of mEHT was not associated with an increased risk in RT delays (OR: 1.614; p = 0.381; 95% CI: 0.553–4.712). Participant tolerance to mEHT treatments was excellent with 95 out of 104 (91.4%) participants receiving eight or more (≥80%) of the planned ten mEHT treatments. The reasons for not receiving more than eight mEHT treatments are adipose burns (n = 2), 1cm blister (n = 1), progressed on treatment (n = 1), moist desquamation resulting in RT and mEHT delay (n = 2), bleeding resulting in RT and mEHT delay (n = 1), did not arrive for RT or mEHT (n = 1), deceased on treatment (n = 1). Two participants in the control group also died during treatment. Eleven (10.6%) mEHT participants reported adverse events: Grade 1–2 adipose tissue burns (n = 10, 9.5%); grade 1 surface burns (n = 2, 2.0%). One participant in the Control Group also reported a subcutaneous burn. A multivariate analysis showed increased BMI (OR: 1.78; p = 0.375; 95% CI: 0.499–6.335), were not predictive of adverse events associated with mEHT treatments.

### Quality of life

At three months post treatment, fatigue and pain were significantly reduced in the mEHT Group and there was a significant improvement in social functioning (p = 0.049), and emotional functioning (p = 0.017) seen in the mEHT Group. There were no significant differences in quality of life between the groups while on treatment.

## Discussion

Our results show that the addition of mEHT to chemoradiotherapy protocols for the management of LACC patients, results in a significantly higher six month LDC and six month LDFS, in a resource-constrained setting and in immunocompromised patients. Hyperthermia is a known radiosensitiser[61] and the heating effect of mEHT during the treatments is therefore likely to be the largest contributor to the improved outcomes seen in patients treated with mEHT in our study. Another possible contributing factor is the immunomodulating effect of mEHT described in the literature which may also contribute to the improved outcomes seen even in patients who are immunocompromised.

Due to resource constraints, the standard of care cisplatin schedule (40mg/m2 weekly), and standard of care of planning, using at least 3D-conformal radiotherapy (3DCRT) and extended fields (para-aortic), where indicated, was not used. It is not clear how much 3DCRT planning and extended radiation fields would improve the results in the treatment groups. Literature indicates that while extended fields on EBRT to include para-aortic lymph nodes significantly reduces the risk of distant metastases and the rate of para-aortic failure, the effects on overall survival are less significant.[57] Resource-constraints also affected factors such as time to complete treatment (listed as technical problems) and timing of the ^18^F-FDG PET/CT scans in some cases (PET/CT down time, source supply problems). The control arm in our group had a lower response than what is generally achieved for LACC patients. Possible reasons include the evaluation of CMR using ^18^F-FDG PET/CT studies compared to clinical evaluations of tumour response, a sample taken from a high risk population many of whom may be immunocompromised, and a resource-constrained setting where patient care and available treatments are sub-standard in comparison to less resource-constrained settings. Under-staging of cervical cancer patients is a well-documented challenge in low-resource settings where imaging techniques are not available.[62] There is a high risk that patients in our setting are being under-staged using only clinical staging methods, compared to the patients staged with imaging studies used in less resource-constrained settings and it is possible that many of our patients are in fact in Stage IV, based on the positive lymph nodes seen on pre-treatment ^18^F-FDG PET/CT scans. The high rate of screening failure was unexpected and we did not expect that the so many of the patients were being under-staged and that there would be so many patients with visceral metastases and bilateral hydronephrosis on PET/CT images. This is an unfortunate challenge in low-resource setting where imaging studies to adequately stage patients are not routinely available.

The reviewed studies on HT for the treatment of cervical cancer, primary or recurrent, use local control, and not local disease control, as an outcome measure and the majority of the studies rely only on the clinical evaluation of local control with only a few using imaging studies.[14–17,20,22–24,63–67] The results from the two Phase III studies on the addition of HT to CRT reported a CR rate of 76% versus 86%(p = 0.54)[23] and 77.6% versus 88%(p = 0.192)[24] in the CRT versus the CRT plus HT groups respectively. In the Harima study[24] CR was achieved when no tumour was detected on physical examination or MRI, evaluated at one month post treatment, although the number of patients evaluated using MRI was not reported. A multivariable logistic regression model did show that patients receiving CRT combined with HT in the Harmia study were more likely to have a CR (*p* = 0.047). Whole pelvis radiotherapy was applied however local disease control was not reported. The difference between this study and our study is the type of capacitive heating used and the frequency of HT treatments per week (one per week in the Harmia study and twice per week in our study). In the Harima study the tumour temperature was monitored rectally and vaginally, not intratumourally, compared to the real-time temperature calculations used by the mEHT device. Although 92% of participants in the Harmia study only received four to six HT treatments, the reasons for not completing HT (for example patient concerns or HT adverse events) are not given[24]. The compliance to mEHT treatments in our study was very good indicating good tolerance of the treatment, with more than 90% receiving eight or more mEHT treatments in our study. In the Phase III study by Flameling et al, the results posted did not include methods of evaluation, distribution of stages of disease, or patient characteristics. The study was closed prematurely due to slow recruitment, and was therefore underpowered, making drawing definitive conclusions impossible. In the Flameling study, HT was administered once per week and the primary tumour response was measured three months post-treatment. [23]

Challenges with clinical staging include the inadequate evaluation of the proximal tumour extension or side wall invasion, and inaccurate lymph node evaluation (especially deep pelvic lymph nodes).[68] With a sensitivity and specificity of lymph node evaluation of 100% and 99.6% respectively for nodes larger than 5mm, and the ability to detect metabolic changes before anatomical changes occur,[69,70] ^18^F-FDG PET/CT is more effective at detecting lymph node involvement,[68] distant metastases,[71] and tumour activity.[72] LDC in our study included lymph nodes within the radiation field as visualised on ^18^F-FDG PET/CT scans and we report on the loco-regional metabolic response, not the clinical response of the tumour.

The major challenges with the management of advanced cervical tumors are loco-regional control and distant metastases. Image guided adaptive brachytherapy (IGABT) has improved local and pelvic control rates for stage III/IVA disease to between 73% and 86%. Pötter et al report that the improved local and pelvic control compared to historical databases is associated with an overall survival benefit of around 10%.[56] These results are significantly higher than those seen in our study. Due to the differences in RT techniques, resources, staffing, patient characteristics, and confounding factors such as malnutrition and immune suppression, seen in our setting compared to less resource-constrained settings, a direct comparison of outcomes between the settings cannot be accurately made.

In our study, BMI and average energy dose were not predictors of six month LDFS, indicating that the mEHT treatments were able to target tumours regardless of the thickness of the adipose layer and that the required temperature and effect is still achieved in patients in whom the applied power needed to be lowered in order to achieve the prescribed treatment duration. In a phase I/II study investigating the effects of HT combined with CRT for the management of 68 LACC participants, adverse events directly attributed to HT included superficial burns (n = 12[18%]) and subcutaneous fatty necrosis (n = 5[7%]).[22] The number of superficial bones in our study was only two, and each was less than 1cm in diameter.

The significance of the results and benefits of HT for the treatment of LACC in the reviewed literature varies considerably. One possible reason is the lack of a fixed reference point for temperature and dose calculations, and the different heating methods.[73] Various heating techniques and protocols (treatment duration, frequency, and timing) are used which may contribute further to the variation in results. The two phase III trials on CRT plus HT which reported no benefit to the addition of HT, used a protocol of one HT treatment per week plus weekly cisplatin (40mg/m2),[23,24] compared to our protocols of two mEHT treatments per week plus two doses of cisplatin (80g/m2) three weekly. Franckena *et al* reported that the number of HT treatments emerged as a predictor of outcomes with patients receiving more than three HT treatments showing a higher CR.[74] Our study did not show an association between the number of mEHT treatments and the CR, however the number of participants with three or less mEHT treatments was only three and the majority (91.3%) of participants could tolerate eight or more mEHT treatments. The need for mEHT treatment discontinuation was very low. Although not all participants could receive cisplatin in our study, the number of cisplatin doses was also not a predictor of outcomes.

Some studies have reported an increase in rates of distant metastases in participants treated with radiation plus HT.[20] We have not observed the same trend. In our study we observed the resolution of extra-pelvic nodal disease along with the pelvic disease in some of the participants. This rate was significantly higher in the mEHT Group. This may be due to the abscopal effect being potentiated by the addition of mEHT. A further analysis of these participants is planned.

According to the literature, treatment outcomes for HIV-positive patients are expected to be worse than in HIV-negative patients.[9–11] However, in our study the HIV-positive participants had a higher rate of complete metabolic tumour response to treatment than the HIV-negative participants, although the difference in CMR between the HIV-positive and -negative participants was not significant (53.3% versus 42.2%; Fisher’s exact: *p = 0*.*340*). In a subgroup analysis of treatment groups in HIV-positive versus HIV-negative participants, the difference between the Control and mEHT Groups was larger in the HIV-negative participants. HIV-positive participants in each treatment group had a higher CMR rate at six months. This may suggest either less of an effect of mEHT in the HIV-positive participants, or a higher radiosensitivity of the tumours in HIV-positive participants. There are indications in the literature that HIV-positive patients have a higher chromosomal radiosensitivity[75] and this may contribute to the increased tumour response rates seen in these patients.

The difference between mEHT and other HT techniques is the dosing concept which relies on the energy dose applied to the patient during the treatment and the temperature is calculated based on the energy dose applied. Intratumoural or MRI thermo-monitoring systems are therefore not required, making the technique far simpler and more affordable than other techniques on the market, enabling the integration of the mEHT into the work flow in the resource-constrained setting. Without the need for increased staff to manage the treatments or the need for access to MRIs, theatres, or specialists, the use of the heating technique was viable. Although the device was loaned to the research team for research purposes, should the department be in a position to purchase the device, the cost to purchase the device is currently less than half the price of a BT device and does not require access to imaging techniques that the more advanced BT systems require. While mEHT is very clearly not a replacement for adequate EBRT and BT planning and treatments, it may be viable technique to improve outcomes in a setting where sophisticated RT techniques are not likely to be available for some time to come. A comparison between our results with mEHT and results from the use of mEHT in a less resource-constrained setting would be merited. Although the response rates varied between our study and the literature, the difference in CR between the two groups in our study is 22% and this improvement in outcomes, even in such high risk patients, is a strong indicator of the potential benefit to the use of mEHT.

## Conclusion

This phase III RCT provides the first evidence for the use of mEHT to improve treatment outcomes when added to CRT for the treatment of LACC. The results offer evidence of efficacy of a heating technique that is feasible and can be implemented in a low resource setting in a population sample in whom treatment and management of LACC is particularly challenging. The results indicate that mEHT can be integrated into the multimodal treatment regimens for LACC patients treated with radiotherapy with/without cisplatin, improving outcomes and potentially assisting in alleviating the burden of LACC on healthcare systems. There is also a potential benefit to the addition of mEHT to CRT for the treatment of LACC in less resource-constrained settings. A longer follow up period is required to assess the survival benefits and further papers are planned to report these results. The design of further phase III studies on mEHT as a chemo- or radiosensitiser for the treatment of other malignancies is warranted. The potential abscopal effect observed suggests that further investigations into the use of mEHT combined with immunotherapies are warranted.

## Supporting information

S1 Fig Reasons for screening failure before randomisation. *Abbreviations*: *VRF*: *Vesicorectal fistula; PET/CT*: *Positron Emission Tomography / Computed Tomography; ARV*: *Antiretroviral* *Bleeding: bleeding that could not be controlled by haemostatic brachytherapy and required admission and a change in protocols before completing the screening process; **Technical: ^18^F-FDG supply difficulties.
(PDF)

S2 Fig Patient BMI versus tumour response on ^18^F-FDG PET/CT. Abbreviations: BMI: Body Mass Index; CMR: Complete Metabolic Response; PMR: Partial Metabolic Response; SMD: Stable Metabolic Disease; PMD: Progressed Metabolic Disease.
(PDF)

S3 Fig Average energy dose versus tumour response on ^18^F-FDG PET/CT. Abbreviations: KJ: Kilojoules; CMR: Complete Metabolic Response; PMR: Partial Metabolic Response; SMD: Stable Metabolic Disease; PMD: Progressed Metabolic Disease.
(PDF)

S1 Table Logistic regression model for prognostic variables and six month local disease free survival. Abbreviations: RT: Radiotherapy; HIV: Human Immunodeficiency Virus.
(DOCX)

S2 Table Logistic regression model of meht-related prognostic variables and six month local disease free survival. Abbreviations: mEHT: Modulated electro-hyperthermia; KJ: Kilojoules; BMI: Body Mass Index
(DOCX)

S1 Appendix CONSORT checklist. (PDF)

S2 Appendix LACC trial protocol. (PDF)
