# Supplementary figures and images for: The effect of modulated electro-hyperthermia on local disease control in HIV-positive and -negative cervical cancer women in South Africa: Early results from a phase III randomised controlled trial
Source: PLoS One. 2019 Jun 19;14(6):e0217894. doi: 10.1371/journal.pone.0217894 (PMC6584021; doi:10.1371/journal.pone.0217894)

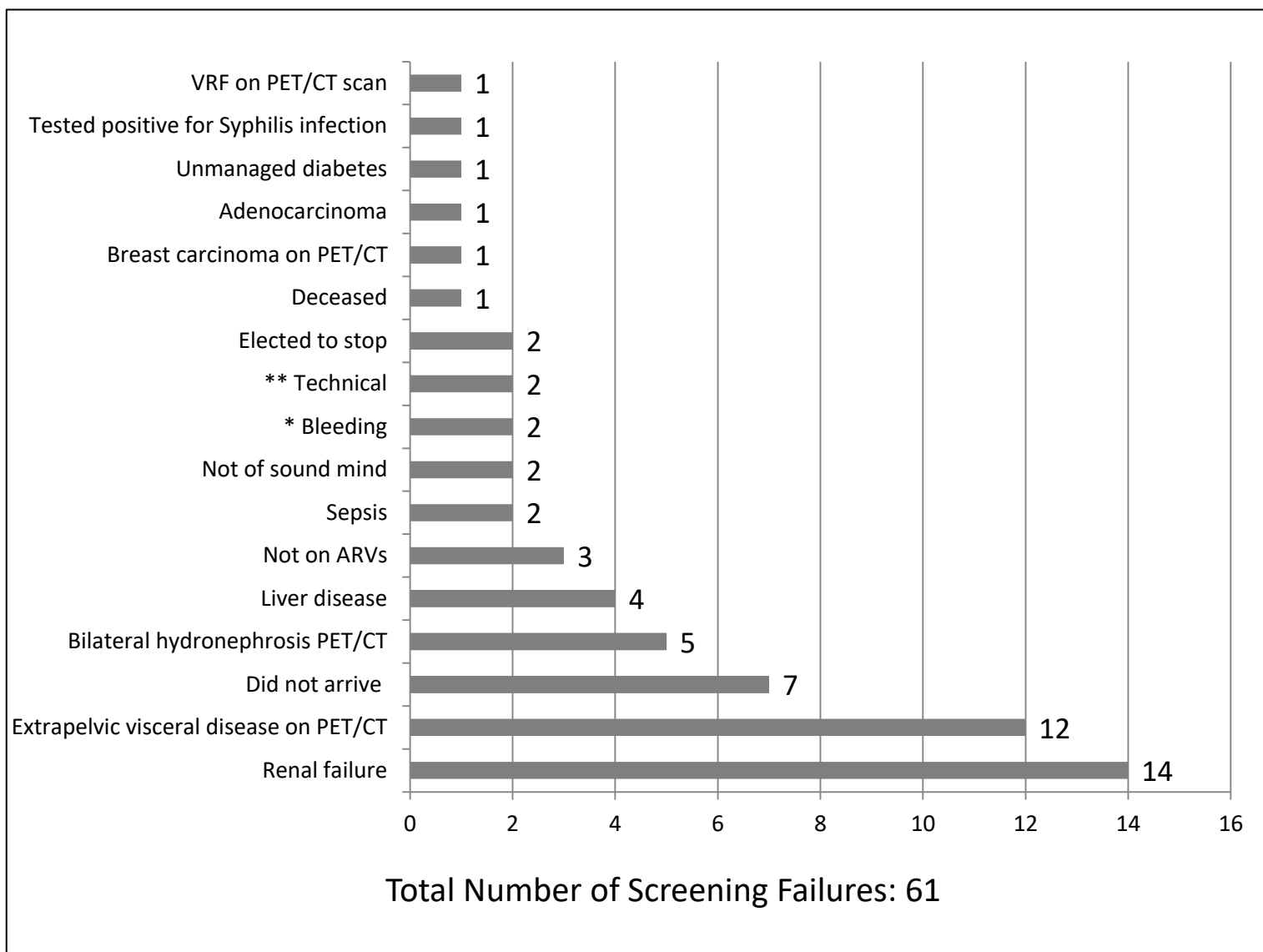

Supplement: S1 Fig — Abbreviations: VRF: Vesicorectal fistula; PET/CT: Positron Emission Tomography / Computed Tomography; ARV: Antiretroviral *Bleeding: bleeding that could not be controlled by haemostatic brachytherapy and required admission and a change in protocols before completing the screening process; **Technical: 18F-FDG supply difficulties. (PDF) [file pone.0217894.s001.pdf]

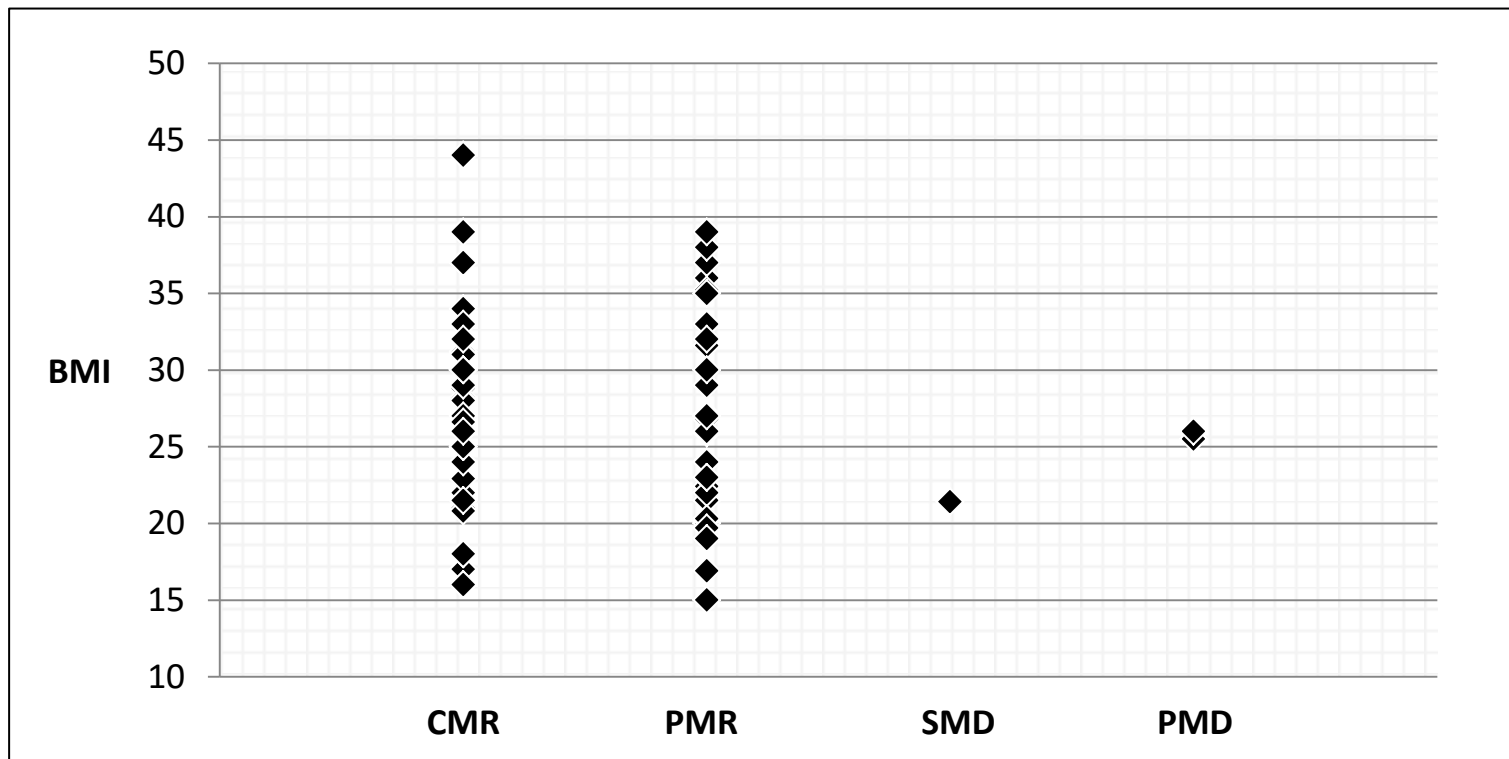

Supplement: S2 Fig — Abbreviations: BMI: Body Mass Index; CMR: Complete Metabolic Response; PMR: Partial Metabolic Response; SMD: Stable Metabolic Disease; PMD: Progressed Metabolic Disease. (PDF) [file pone.0217894.s002.pdf]

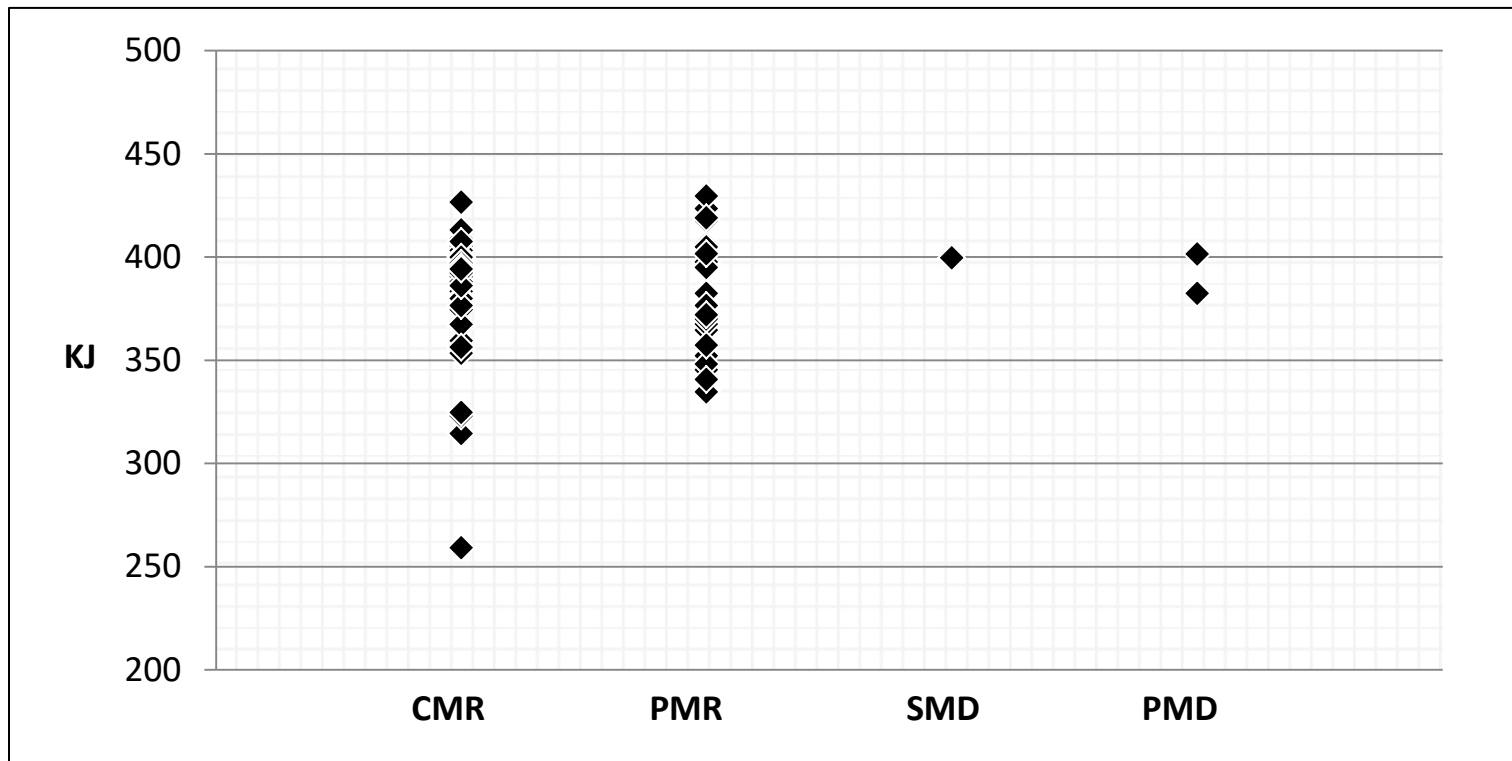

Supplement: S3 Fig — Abbreviations: KJ: Kilojoules; CMR: Complete Metabolic Response; PMR: Partial Metabolic Response; SMD: Stable Metabolic Disease; PMD: Progressed Metabolic Disease. (PDF) [file pone.0217894.s003.pdf]
