# Supplementary material for: The effect of modulated electro-hyperthermia on local disease control in HIV-positive and -negative cervical cancer women in South Africa: Early results from a phase III randomised controlled trial
Source: PLoS One. 2019 Jun 19;14(6):e0217894. doi: 10.1371/journal.pone.0217894 (PMC6584021; doi:10.1371/journal.pone.0217894)
Supplement: S1 Table — Abbreviations: RT: Radiotherapy; HIV: Human Immunodeficiency Virus. (DOCX) [file pone.0217894.s004.docx]

| Prognostic Variable | Odds Ratio  [OR] | P>\|z\| | 95% Confidence interval [CI] range |
| --- | --- | --- | --- |
| *All Participants (multivariate)* | | | |
| Age | 1.03 | 0.064 | 0.993-1.060 |
| Number of cisplatin doses | 1.14 | 0.578 | 0.72-1.80 |
| Overall treatment time | 0.98 | 0.448 | 0.93-1.03 |
| Total RT dose | 1.05 | 0.247 | 0.96-1.15 |
| *HIV-positive participants only (univariate)* | | | |
| CD4 count | 1.00 | 0.279 | 0.999-1.003 |
