# Supplementary material for: The effect of modulated electro-hyperthermia on local disease control in HIV-positive and -negative cervical cancer women in South Africa: Early results from a phase III randomised controlled trial
Source: PLoS One. 2019 Jun 19;14(6):e0217894. doi: 10.1371/journal.pone.0217894 (PMC6584021; doi:10.1371/journal.pone.0217894)
Supplement: S2 Table — Abbreviations: mEHT: Modulated electro-hyperthermia; KJ: Kilojoules; BMI: Body Mass Index (DOCX) [file pone.0217894.s005.docx]

| Prognostic Variable | Odds Ratio  [OR] | P>\|z\| | 95% Confidence interval [CI] range |
| --- | --- | --- | --- |
| No of mEHT treatments | 1.165 | 0.359 | 0.84-1.62 |
| Energy Dose (KJ) | 0.991 | 0.075 | 0.98=1.00 |
| BMI | 0.995 | 0.886 | 0.93-1.06 |
