# Supplementary material for: The effect of modulated electro-hyperthermia on local disease control in HIV-positive and -negative cervical cancer women in South Africa: Early results from a phase III randomised controlled trial
Source: PLoS One. 2019 Jun 19;14(6):e0217894. doi: 10.1371/journal.pone.0217894 (PMC6584021; doi:10.1371/journal.pone.0217894)
Supplement: S2 Appendix — (PDF) [file pone.0217894.s007.pdf]

THIS PROTOCOL WAS UPDATED AND SUBMITTED TO ETHICS IN APRIL 2017 T0 APPLY FOR AN EXTENSION OF TWO YEARS. INVESTIGATOR NAMES ALSO WERE UPDATED.

Trial Protocol

A phase III randomised trial investigating the benefits of the addition of Modulated electro-hyperthermia to chemo-radiation for cervical cancer in HIV positive and negative women in South Africa

2012

**Sponsors:**	National Research Foundation	
**Therapy:**	Modulated electro-hyperthermia<br>Radiation therapy<br>Chemotherapy	
**Principal Investigators:**	Jeffrey Kotzen (Supervisor)<br>Carrie Minnaar (PhD Candidate)	
**Co-Investigators:**	Radiation oncology	Mariza Tunmer<br>Thanushree Naidoo<br>Vinay Sharma
	Nuclear medicine	Olusegun Akinwale Ayeni<br>Mboyo-Di-Tamba Vangu
	Radiobiology	Ans Baeyens (Co-supervisor)

TABLE OF CONTENTS

TABLE OF CONTENTS I

LIST OF ABBREVIATIONS V

STUDY SYNOPSIS VI

1. INTRODUCTION 1

1.1. Hyperthermia in Oncology 1
1.2. Current Treatment for Cervical Cancer in South Africa 1
1.3. Study Rationale 1
1.4. Hypotheses 2

2. STUDY OBJECTIVES 3

2.1. Primary Objectives 3
2.2. Secondary Objectives 3
2.3. Exploratory Objectives 3

3. PARTICIPANT SELECTION 4

3.1. Inclusion Criteria 4
3.2. Exclusion Criteria 4
3.3. Number of Participants to be Enrolled 5
3.4. Recruitment Procedures 5
3.5. Enrolment Procedures 6
3.6. Randomisation 6

4. STUDY MODALITIES 7

4.1. Diagnosis and Staging 7
4.2. Treatment Summary 7
4.2.1. Chemotherapy 7
4.2.2. Radiation Therapy 7
4.2.3. Modulated electro-hyperthermia 8
4.2.4. Antiretroviral Therapy (ARV) 8
4.3. Duration of Study 8
4.4. Follow-up Appointments and Monitoring of Participants 8

5. TREATMENTS 9

5.1. Cisplatin 9

5.1.1. Dosage ... 9
5.1.2. Adverse Effects ... 9
5.1.3. Supplier ... 9
5.2. Radiation Therapy ... 9
5.2.1. Emergency Radiation Therapy to Stop Bleeding Prior to Treatment ... 9
5.2.2. External Irradiation ... 9
5.2.3. Intracavitary Brachytherapy ... 10
5.2.4. Dose Distribution for All Regimens ... 10
5.2.5. Radiation Equipment ... 10
5.2.6. Radiation Therapy Quality Control and Documentation ... 10
5.2.7. Radiation Toxicity ... 10
5.2.8. General Radiation Schedule ... 11
5.3. Modulated electro-hyperthermia ... 11
5.3.1. Dose ... 11
5.3.2. Administration ... 12
5.3.3. Personnel ... 12
5.3.4. Equipment ... 12
5.3.5. Quality Control and Documentation ... 12
5.3.6. Expected Toxicity ... 12
5.3.7. General Modulated electro-hyperthermia Schedule ... 13
6. TREATMENT MODIFICATIONS ... 14
6.1. Cisplatin ... 14
6.1.1. Cisplatin Modifications ... 14
6.1.2. Completion of Chemotherapy ... 16
6.2. Radiation Therapy ... 16
6.2.1. Radiation Dose Modifications ... 16
6.2.2. Dose Delays ... 17
7. ADVERSE EVENTS MONITORING AND REPORTING ... 18
7.1. Classification of Adverse Events ... 18
7.1.1. Toxicity ... 18
7.1.2. Expectedness ... 18
7.1.3. Attribution ... 18
7.2. Reporting Procedures ... 19
7.2.1. Routine AE Reporting ... 19
7.2.2. Grade 2 and 3 AEs ... 19
7.3. Modulated electro-hyperthermia ... 19
8. OUTCOME MEASURES ... 20
8.1. Primary Outcome Measures ... 20
8.1.1. Local Disease Control ... 20
8.1.2. Progression Free Survival ... 20
8.1.3. Two Year Survival ... 20

8.2. Secondary Outcome Measures 20
8.2.1. Safety and Tolerability 20
8.2.2. Quality of life 21
8.2.3. Cost per life 21
8.2.4. Effects of treatment on the participant's HIV disease status 21
8.2.5. Recurrence 22
8.3. Exploratory Outcome Measures 22
9. CLINICAL AND LABORATORY EVALUATIONS 23
9.1. Screening Evaluations 23
9.1.1. Within 14 days prior to initiating protocol therapy 23
9.2. Evaluations during Chemo-Radiation Treatment 23
9.2.1. During Chemo-Radiation 23
9.2.2. During Brachytherapy 23
9.3. Evaluations after Completion of Treatment Protocols 23
9.3.1. 3 Months after completion of treatment 23
9.3.2. 6 Months after completion of treatment 24
9.3.3. 9 Months after completion of treatment 24
9.3.4. 12 Months after completion of treatment 24
9.3.5. 18 Months after completion of treatment 24
9.3.6. 24 Months after completion of treatment 24
9.4. Evaluation of Recurrence 24
9.5. Off Study Evaluation 25
10. DATA MANAGEMENT 26
10.1. Data Quality 26
10.2. Role of Data Manager 26
11. STATISTICAL ANALYSIS 27
11.1. Outcome variables 27
11.2. Tumour characteristics 28
11.3. Participant characteristics 28
11.4. Sample Size Considerations 28
11.5. Site(s) of Recurrence 28
11.6. Toxicity Analysis 28
11.7. Statistical Analysis Plan 29
12. EXPLORATORY RESEARCH 30
13. ETHICAL AND REGULATORY CONSIDERATIONS 31
13.1. Informed Consent (see APPENDIX G and H) 31

13.2. Changes to the Protocol 31
13.3. Women and Minorities 31
13.4. Subject Confidentiality 31
13.5. Study Discontinuation 32

APPENDIX A QUALITY OF LIFE QUESTIONNAIRE 33

APPENDIX B ECOG 34

APPENDIX C BODY MASS INDEX TABLE 35

APPENDIX D ELIGIBILITY CHECKLIST AND ENROLMENT FORM 36

APPENDIX E INFORMATION AND CONSENT FORM 39

APPENDIX F EXPLORATORY INFORMED CONSENT FORMS 48

APPENDIX G FIGO STAGING CRITERIA 50

APPENDIX H RESOURCE SHEET 51

APPENDIX I ADVERSE EVENTS 52

REFERENCES 53

LIST OF ABBREVIATIONS

AE	Adverse Event
ALT	Alanine Aminotransferase
ANC	Absolute Neutrophil Count
ART	Antiretroviral Therapy
AST	Aspartate Aminotransferase
ATP	Adenosine Triphosphate
BMI	Body Mass Index
CT	Computer Tomography
CTC	Common Toxicity Criteria
DNA	Deoxyribonucleic Acid
ECM	Extracellular Matrix
ECOG	Eastern Cooperative Oncology Group
EHT	Electro-Hyperthermia
FIGO	International Federation of Gynaecology and Obstetrics
EORTC	European Organisation for Research and Treatment of Cancer
HDR	High Dose Rate
HRCT	Hyperthermia plus Radiation therapy plus Chemotherapy
HSPs	Heat Shock Proteins
HT	Hyperthermia
IV	Intravenous
LACC	Locally Advanced Cervical Carcinoma
LDC	Local Disease Control
mEHT	Modulated Electro-Hyperthermia
OS	Overall Survival
OT	Modulated electro-hyperthermia
PAP	Papanicolaou
PFS	Progression-Free Survival
QOL	Quality of Life
RCT	Radiation therapy plus Chemotherapy
RHT	Radiation therapy plus Hyperthermia
RNA	Ribonucleic Acid
RT	Radiation Therapy

STUDY SYNOPSIS

**DESIGN**	Randomised, Phase III Clinical trial
**DURATION**	72 months (48 months of recruitment and 24 months of follow-up)<br>*Increased 48 months based on slowerr recruitment than anticipated*
**SAMPLE SIZE**	267 women will be enrolled and randomly assigned to the study or the control arm of the trial *(increased from 236 to 267 due to higher than expected screening failure rate)*
**POPULATION**	HIV negative and HIV positive women with locally-advanced cancer of the cervix, Stages IIB (distil) to III.
**REGIMEN:**	All participants will receive 50Gy external beam radiation, 3 x 8Gy HDR brachytherapy treatments, and two doses of chemotherapy (Cisplatin) three weekly – subject to dose modification rules – administered during the radiation therapy. The women in the study group will receive two modulated electro-hyperthermia treatments per week during the radiation treatment.
**PRIMARY OBJECTIVES**	1) To determine the local disease control after treatment at 6 months<br>2) To determine the progression-free survival (PFS) at 6, 12, 18 and 24 months after the last treatment date.<br>a) Determine PFS in all registered participants, regardless of completion (Intent To Treat-ITT)<br>b) Determine PFS in the subset of participants who complete the prescribed chemo-radiotherapy<br>3) Determine the overall survival at two years
**SECONDARY OBJECTIVES**	1) To evaluate the adverse events associated with modulated electro-hyperthermia.<br>2) To evaluate the effect of modulated electro-hyperthermia on chemotherapy and RT tolerability and toxicity.<br>3) To describe the quality of life of enrolled participants via assessments before, at 6 weeks, and at 3, 6, 9, 12, 18 and 24 months after completion of therapy using a Quality of Life (QOL) questionnaire (see APPENDIX A)<br>4) To evaluate economic viability of the addition of modulated electro-hyperthermia to standard treatment protocols<br>5) To evaluate the effect, if any, of modulated electro-hyperthermia treatments on the HIV disease status of HIV positive patients, assessed by presence of AIDS defining illnesses before and after treatment.<br>6) To describe and compare cervical cancer recurrence patterns in all participants
**EXPLORATORY RESEARCH**	1) To evaluate thermoradiosensitivity by measuring DNA damage using Micronucleus (MN) assay before and after completing treatment.<br>2) To compare gene expression profiles of lymphocytes and cervical epithelial cells extracted from biopsies before and after modulated electro-hyperthermia treatment

1. INTRODUCTION

1.1. Hyperthermia in Oncology

The benefits of hyperthermia (HT) treatments in combination with chemotherapy and radiation therapy (RT) have been demonstrated in a number of trials conducted on a wide variety of tumours. [1, 2] Studies have shown that the increased sensitivity to radiation and chemotherapy damage as a result of HT helps to improve local disease control (LDC). Improved local disease control may improve quality of life (QOL) and increase overall survival (OS). The potential for this to reduce healthcare costs has also been demonstrated.[3, 4, 5]

The technical difficulties, costs, adverse effects and the lack of standardisation and dose control of conventional HT techniques have resulted in mixed opinions on the use of HT in oncology practices. Oncotherm GmbH (Germany), established in 1988, has resolved these difficulties with the development of modulated electro-hyperthermia (EHT) devices. The characteristics of the Oncotherm devices make the technology feasible for use in a low resource setting.

The clinical value of HT in addition to other treatment modalities has been shown in randomised phase II and III trials conducted primarily in Europe and America. Significant improvements in clinical outcome have been demonstrated for tumours of the head and neck, melanoma, breast, brain, bladder, cervix, rectum, lung, gastrointestinal tract, vulva, vagina and soft tissue sarcomas when treated with HT in combination with standard therapies. [2]

The body of research on the use of HT for the treatment of cervical cancer is growing rapidly with numerous phase I and II studies having demonstrated safety and efficacy of the addition of HT to chemo-radiation and radiation alone for the treatment of cervical cancer. [6, 3, 7, 8, 9, 10, 11, 12, 13, 14, 15]

1.2. Current Treatment for Cervical Cancer in South Africa

The standard treatment for LACC is concurrent chemoradiotherapy, using cisplatin as the chemotherapeutic radiosensitising agent. Multiple randomised controlled-trials have demonstrated improved efficacy when cisplatin was added to RT. The addition of cisplatin increases the risk of haematological, renal and gastrointestinal toxicities and is contraindicated in HIV positive patients with a CD4 count below 200/µL.

1.3. Study Rationale

The African setting is unique in that the numbers of patients and stages of disease are high and the resources and funding are limited. The potential cost per life saved resulting from the potential increase in treatment efficacy by the addition of HT could significantly lower the burden on healthcare facilities in South Africa and Africa. Despite the potential benefits, there have to date, been no trials investigating HT or mEHT in Africa.

The large incidence of HIV in Africa compared to developed countries in which HT trials have been conducted also poses a unique situation. The HIV status of a patient affects their immune function and chemotherapy and radiation toxicity. Authors have suggested that HT may be beneficial to patients in whom treatment doses must be lowered due to dose limiting toxicities. HT and mEHT, may therefore have a role in improving treatment outcomes in HIV positive patients in whom treatment doses must be lowered.

This trial will be the first of its kind for a number of reasons and the results have the potential to significantly enhance the current available treatments in South Africa:

- This will be the first trial on HT and its benefits and role in an African setting.
- The trial will also be the first to evaluate the effects of HT on HIV positive oncology patients.
- This will be the first phase III trial on this particular type of hyperthermia.
- The economic evaluation will evaluate the feasibility of the technology in Africa.
- The trial will be comprehensive, taking into account all factors associated with HT, such as treatment toxicity, adverse effects and quality of life.
- Previous trials have described the effects of HT on the DNA. The exploratory research conducted on the tumour samples taken from all groups in this trial before and after treatment will further add to this body of research.
- Previous phase III trials on hyperthermia in cervical carcinoma patients have focussed on HT and RT. We will be including chemotherapy in the protocols making this one of the first phase III trials to evaluate the trimodality treatment of HT, RT and chemotherapy.

1.4. Hypotheses

1) The addition of mEHT to RCT protocols in LACC participants will result in improved local disease control.
2) An improved 2 year disease free survival will be seen in the groups receiving RCT combined with mEHT.
3) The use of mEHT in HIV positive participants will improve the 2 year disease free survival even in participants in whom treatment doses must be lowered due to dose limiting toxicities.
4) The addition of mEHT will not significantly increase the toxicity of chemotherapy or RT.
5) The adverse effects of mEHT will be uncommon and manageable.
6) The quality of life in the group receiving mEHT will be better than the group not receiving mEHT.
7) The DNA damage to the tumours of participants in the group receiving mEHT will be more significant than in the group not receiving mEHT.

2. STUDY OBJECTIVES

2.1. Primary Objectives

1) To evaluate the local disease control at 6 months in HIV positive and HIV negative participants with stage IIB (locally advanced) to III cervical cancer after five weeks of external beam RT, three doses of HDR brachytherapy and cisplatin either alone or combined with mEHT.
2) To determine the progression-free survival (PFS) at 6, 12, 18 and 24 months after last treatment date of HIV negative and HIV positive stage IIB (locally advanced) to III cervical cancer participants receiving either chemoradiation alone or in combination with mEHT.
    a. Determine PFS in all registered participants, regardless of their completion (Intent To Treat-ITT)
    b. Determine PFS in the subset of participants who complete the prescribed RCT with or without mEHT treatments.
3) Determine the overall survival at two years and the cause of death (i.e. cancer-related, HIV-related, treatment related or other).

2.2. Secondary Objectives

1) To evaluate the adverse events that can be directly attributed to mEHT treatments.
2) To evaluate the effects of mEHT on tolerability and toxicity of the prescribed RCT treatments.
    a. Evaluate the number of dose delays for chemotherapy and the reasons for the delayed dose administration in both groups.
    b. Evaluate the number and causes of the dose reductions of chemotherapy in both groups.
    c. Evaluate the number of missed radiation treatments and the reasons for the missed treatments in all four groups.
    d. Evaluate the number and causes of the dose reductions of radiation in both groups.
    e. Evaluate the early and late complications associated with the prescribed treatments.
3) To describe the Quality of Life (QOL) of enrolled participants via assessments before, 6 weeks after, and at 3, 6, 9, 12, 18 and 24 months after completion of therapy using a QOL questionnaire and a visual analogue scale (see APPENDIX A)
4) To evaluate the economic implications of treatments per life per year in the two groups in order to assess the economic benefits of the addition of mEHT to standard treatment protocols and the viability of the treatment in South African healthcare settings.
5) To evaluate the effect, if any, of mEHT treatments on the HIV disease status of HIV positive participants:
    a. CD4 count
    b. HIV viral load
    c. Concurrent AIDS-defining conditions
6) To describe cervical cancer recurrence patterns in both groups
    a. Loco-regional and distant recurrences
    b. Recurrence patterns by initial stage and suspicion for nodal metastasis pre-treatment

2.3. Exploratory Objectives

1) To evaluate thermoradiosensitivity by measuring DNA damage using Micronucleus (MN) assay before and after completing treatment
2) To compare gene expression profiles of lymphocytes and cervical epithelial cells extracted from biopsies before and after mEHT treatment

3. PARTICIPANT SELECTION

3.1. Inclusion Criteria

1) Participants (who have been adequately clinically staged by standard clinical guidelines) with biopsy proven primary, untreated, histologically confirmed invasive squamous and aden-squamous cell carcinoma of the uterine cervix, FIGO stages advanced IIB (invasion of the distal half of the parametrium), IIIA and IIIB.
2) HIV positive participants will be accepted.
3) The following laboratory tests will be done prior to enrolment in the study and the values must be in the following ranges:
   - Haemoglobin >10 g/dL;
   - Platelet count >150/mm^3;
   - ANC >3000/mm^3
   - Creatinine clearance>60 mL/min
   - Liver function tests
4) Females over the age of 18 years
5) Ability to understand and the willingness to sign a written informed consent document.
6) Eastern Cooperative Oncology Group (ECOG) score of not more than 2 (see APPENDIX B).
7) Participants of childbearing potential must have a negative urine or serum pregnancy test prior to enrolment and use an effective form of contraception (e.g. barrier contraception, highly effective hormonal contraception).
8) At the investigators' discretion, participants must be suitable for treatment with radical intent using concurrent chemotherapy and pelvic radiation. Subjects who undergo emergency RT in the form of brachytherapy for haemostasis, prior to enrolment will be allowed to be screened and enrolled provided they meet all other eligibility criteria.
9) Life expectancy of greater than 12 months.
10) Participants must have a body mass index (BMI) that is within normal ranges (see APPENDIX C).

3.2. Exclusion Criteria

1) Participants who have undergone hysterectomy.
2) Patients with life-threatening AIDS defining illnesses (other than cervical carcinoma) will be excluded, as will patients with a CD4 count < 200/µL and not on ARVs.
3) Patients with acute active (such as tuberculosis or malaria), serious, uncontrolled infections will be excluded.
4) Participants will be excluded if there is evidence of resistance to antiretroviral therapy (i.e. HIV viral load $\geq$ 400 copies/mL despite combination antiretroviral therapy for at least 4 months).
5) Prior invasive malignancy other than LACC diagnosed within the past 24 months, excluding in situ anal dysplasia or carcinoma in situ, non-melanoma skin carcinoma, or Kaposi's sarcoma that has not required systemic chemotherapy within the past 24 months.
6) Pregnant or breast-feeding women.
7) A medical or psychiatric illness that prevents the participant from being able to sign an informed consent or would affect the participant's ability to comply with the protocol stipulations.
8) Participants with circumstances that will not permit completion of the study or required follow-ups. For instance if travel to and from treatment site is an issue.
9) Participants with carcinoma of the cervical stump.

10) Participants with a history of cardiovascular disease manifested as
    a) History of myocardial infarction
    b) Unstable angina
    c) Currently taking medication for treatment of angina
    d) History of coronary artery bypass surgery
11) Participants with contraindications to modulated electro-hyperthermia treatment:
    a) Pace makers and other implanted devices which rely on current and charges.
    b) Large metal implants, such as hip replacements.
    c) Inability to feel temperature in the region.
    d) Inability to express or vocalise discomfort or heat at the treatment site.
12) Exclude vesicovaginal and vesicorectal fistulas, extra-pelvic visceral metastases and bilateral hydronephrosis on ^{18}F-FDG PET/CT

3.3. Number of Participants to be Enrolled

This study will aim to enrol a total of 236 participants (N1=118, N2=118).* This is based on the estimated required sample sizes for a two-sample comparison of survivors' functions at two years. We estimate an expected reduction in mortality of 50%, (based on survival of 20% in the control group and 40% in the experimental group). The statistical significance is defined as a two-sided alpha<0.05 for a log-rank test, with a constant Hazard ratio of 0.5693, a statistical power of 90%, a 15% withdrawal rate and an estimated 140 events.

It is estimated that around 30% of these participants will be HIV positive. Participants will be randomly assigned into two groups of 118 to receive either RCT alone or in combination with mEHT. We anticipate an accrual rate of five participants per month. Recruitment is therefore expected to take an estimated forty eight months.** All participants will have a minimum of two years of follow-up. We anticipate at least 50% of recruited participants will be in Stage III of the disease. The participant numbers will be re-evaluated once 150 participants have been enrolled and the decision will be taken to either continue to enrol a further 88 participants or to utilise the results of the 150 participants already enrolled in the study. The decision will be based on preliminary results which will be used to draw a more accurate conclusion of the number of participants required in order to maintain a 90% statistical power.

*Increased to 271 participants due to higher than expected screening failure
** Increased to 72 months due to slower than anticipated recruitment

3.4. Recruitment Procedures

The study will be conducted at the Charlotte Maxeke Johannesburg Academic Hospital in Gauteng, South Africa, by the radiation oncology department at the University of the Witwatersrand. Permission to conduct the trial at the hospital has been obtained from the Chief Executive Officer (APPENDIX D), with approval from the Human Research Ethics Committee. APPENDIX E confirms approval for the research to be done at the radiation oncology department at the University of the Witwatersrand. Participants who, after evaluation, diagnosis and screening, have been found by their treating doctor, to be eligible for the trial will be approached to voluntarily participate in the study. Patient eligibility will be based on the eligibility checklist (see APPENDIX F) which contains all inclusion and exclusion criteria. Eligible participants will be asked if they would like information on the trial. Patients who would like information will be given an Information and Consent Form (APPENDIX G) to read through and will be have the information sheet clearly explained to them in a language the patients are comfortable with. Patients will be given another information and consent form for the exploratory research (APPENDIX H), should they wish to participate.

3.5. Enrolment Procedures

Once the participant is comfortable with the informed consent form and confirms that she has understood the contents clearly, she will be asked if she would like to participate in the trial. Patients who agree to participate will be asked to sign a consent form which will give permission to the researchers to access the participant's medical information including all routine tests and investigations pertaining to the patient's current health and diagnosis as well as all subsequent medical records. Upon signature of the informed consent the participants will be asked to complete a Quality of Life questionnaire with a visual analogue scale. Participants will then be booked for study specific investigations – PET/CT.

All enrolment forms will be numbered and the number that appears on the enrolment form will be the reference number assigned to the participant for the duration of the trial. This number will replace the participant's personal details on all the documents pertaining to the participant that are used for the trial.

3.6. Randomisation

Once the participant has her study reference number for the trial, she will be randomly assigned to receive either RCT alone or in combination with mEHT. The sampling method used will be stratified random sampling (stratum: HIV status). In each stratum there will be a random selection.

4. STUDY MODALITIES

4.1. Diagnosis and Staging

Participants must have histologically confirmed invasive squamous cell or adeno-squamous carcinoma of the cervix. Diagnosis should be done using a representative H & E stained slide. Copies of histology and laboratory reports are to be given to the data manager for confirmation of diagnosis and storage and to be entered into the data management system.

Participants must have undergone clinical staging by physicians according to FIGO staging criteria (See APPENDIX I). In order to correctly stage the participants and to determine eligibility the following investigations must also be done:

- Chest x-ray
- Ultrasound (in order to rule out hydronephrosis)
- Physical assessment

4.2. Treatment Summary

All participants with cervical carcinoma confined to the pelvis (Stages IIB to III) will receive pelvic radiation, brachytherapy and concurrent cisplatin as outlined below. Participants in the study arms will receive pelvic radiation, brachytherapy and concurrent cisplatin as well as local mEHT treatments. Participants in the control arm will receive only pelvic radiation, brachytherapy and concurrent cisplatin. The administration of all prescribed concomitant medications, such as anti-emetics, anti-nausea, anti-diarrhoea etc., must be documented in the participant's and recorded by the data management team.

4.2.1. Chemotherapy

All participants will receive treatment with the platinum based chemotherapy: Cisplatin

- Three doses of 80 mg/m2 administered twenty one days apart during the five weeks of RT
- To be administered intravenously (IV) over 30-60 minutes
- Administered on days 1, 21 of external beam RT treatment

4.2.2. Radiation Therapy

All participants will receive RT in the form of external beam radiation and high dose rate (HDR) brachytherapy.

1) External Beam Radiation:
   - A dose of 50.0 Gy over five weeks administered to the pelvis
   - 2 Gy per fraction at the Iso centre
   - 25 fractions

2) HDR Brachytherapy
   - 24 Gy intracavitary brachytherapy
   - Administered in 8 Gy per dose to point A
   - Total of 3 HDR doses

4.2.3. Modulated electro-hyperthermia

Participants in the study groups will receive bi-weekly mEHT treatments for fifty five minutes at 130 W over and above the prescribed RCT.

4.2.4. Antiretroviral Therapy (ARV)

HIV positive patients must be enrolled at an HIV clinic and must be receiving combination antiretroviral therapy with an acceptable regimen that adheres to national guidelines for the treatment of HIV infection. Participants who are already on a regime prior to entering the study may remain on the same regime. HIV monitoring and ARV administration will be done at the HIV clinic and results and confirmation of treatment regimens will be requested, with the participant's permission, from the clinic by the investigating doctors.

4.3. Duration of Study

As each participant in the trial must have a life expectancy of at least twelve months, it is expected that the majority of participants would be able to complete the prescribed treatment schedule.

A total of 236 participants will be enrolled at a rate of approximately 5 participants per month. Total enrolment time is therefore estimated to take forty eight months.

Follow up consultations and monitoring of each participant who has completed the treatment protocol will be done over a two year period. In cases where participants are unable to complete the treatment regime, either due to treatment toxicity, disease progression or other reasons, participants will still be followed up every six months and monitored for two years.

The total trial duration is therefore expected to take six years.

4.4. Follow-up Appointments and Monitoring of Participants

Participants will be contacted one month prior to the follow up date in order to confirm a date and time for the follow up. The communication must be done in a friendly and encouraging manner using a means of communication and language that is suitable for the participant. Participants will also be reminded of the appointment the week prior to the date and the day before the appointment. The reminders may be done via text messages or phone calls.

Although participants will be encouraged to attend follow-up visits. Should they decide not to attend the visits or are unable to attend the follow-ups then the staff may obtain information regarding the participant's general health, adverse events, medication changes, QOL survey or other appropriate changes verbally. This information must be documented within the participant's medical.

5. TREATMENTS

5.1. Cisplatin

5.1.1. Dosage

Two doses of three weekly 80mg/m2 of cisplatin will be administered to all participants, subject to the stopping rules described in section 6.1. The clearance will be calculated within four days of chemotherapy administration.

5.1.2. Adverse Effects

1) Common
Allergic reactions, haematological (leucopoenia, thrombocytopoenia, anaemia), gastrointestinal (nausea, vomiting), nephrotoxicity, electrolyte imbalance, hypocalcaemia, hypomagnesaemia, ototoxicity, aminoglycoside ototoxicity, ocular toxicity and peripheral neuropathy

2) Infrequent
Cardiac abnormalities, anorexia, elevated AST, rash, alopoecia and acute myeloid leukaemia.

3) Renal Toxicity
Mild renal dysfunction is a common complication. Aminoglycoside antibiotics given before, with, or after cisplatin may potentiate renal toxicity and should be avoided whenever possible.

5.1.3. Supplier

To be purchased from commercial sources.

*Refer to Package Insert for additional information.

5.2. Radiation Therapy

5.2.1. Emergency Radiation Therapy to Stop Bleeding Prior to Treatment

In the event that emergency radiation is required to stop the bleeding of tumours prior to commencing the treatment protocols, participants will still be allowed to enter the study provided all other eligibility criteria are still met. Radiation treatment should be started as soon as possible after the administration of emergency radiation so the total treatment time does not exceed 56 days.

5.2.2. External Irradiation

All participants will receive 50 Gy external beam RT delivered homogeneously to the pelvis in 25 fractions of 2 Gy. Fractions will be administered daily, five days a week, over a period of five weeks.

The dose to the pelvis shall be calculated at the intersection of the axes of the 4-field box. The dose to point B shall also be calculated using an off-axis calculation (or identified on the isodose distribution).

The dose to central structures shall be the sum of the dose at the intersection of the axes of the 4-field box and the dose to point A from the intracavitary treatment. The dose to side wall structures shall be

calculated bilaterally and shall be the sum of the dose at the intersection of the axes from the 4-field box plus the intracavitary dose to each point B plus the contribution from the parametrial fields.

- Fractionation: Conventional fractionation will consist of one fraction per day, total five fractions per week.
- Therapy interruptions: If interruption of radiation should occur for greater than two weeks, the Principal Investigators must be notified.

5.2.3. Intracavitary Brachytherapy

High Dose Rate (HDR) brachytherapy will be administered in three doses of 8 Gy fractions.

1) Timing

HDR brachytherapy should be started in the third week of external RT treatment. When HDR brachytherapy begins, at least one insertion will be performed per week with no external beam therapy given on the day of the insertion. If the majority of the external beam radiation has been given, then two insertions per week could be done separated by at least 72 hours in order to complete all treatment within eight weeks.

2) Doses

Each HDR brachytherapy fraction will give 8 Gy to Point A. Each participant will be treated with three insertions of 8.0 Gy/fraction to deliver a total of 24 Gy.

3) HDR Instruments

Tandem or rings will be used for HDR brachytherapy.

5.2.4. Dose Distribution for All Regimens

A four-field box technique with parallel opposed AP/PA and two opposing lateral fields should be used. The dose distribution across the clinical target volume should not vary more than 5% from the recommended dose and all fields must be treated daily.

5.2.5. Radiation Equipment

All participants will undergo simulation for localisation, and verification of external RT treatment portals. CT scan without contrast is required in pre-treatment planning. Orthogonal films should be performed for each implant. Intra-cavity treatment will be delivered by iridium for HDR in tandem or ring applicators.

5.2.6. Radiation Therapy Quality Control and Documentation

Standard department Quality Assurance protocols for the radiation department at The Charlotte Maxeke Johannesburg Academic Hospital will apply for this study.

5.2.7. Radiation Toxicity

1) Haematologic Toxicity
Haematologic toxicity is an uncommon symptom of pelvic radiation toxicity, unless the RT is combined with chemotherapy. Haematological toxicities may include immune suppression, thrombocytopoenia and low haemoglobin levels.

2) Gastrointestinal Toxicity

Gastrointestinal symptoms are the most likely adverse effects resulting from RT to the pelvis. Although nausea and vomiting are uncommon, these symptoms may occur after the first few treatments. In the event that participants report nausea, anti-emetics should be administered. Severe, prolonged and unmanageable nausea and vomiting warrants further investigations to rule out recurrent tumours or other causes of bowel obstruction.

Increased bowel activity with diarrhoea can be expected after the first two weeks of pelvic radiation. Participants should be advised to follow a low fibre, low fat and bland diet in order to reduce the diarrhoea. Anti-diarrhoeal medications are likely to be required by most participants.

In severe cases participants may require hospitalisation in order to manage the gastrointestinal symptoms and to administer intravenous fluids.

3) Renal/Genitourinary Toxicity

Radiation to the pelvis may result in acute toxicity to the urinary tract. The most common manifestation of urinary toxicity is cystitis. In the event that haematuria develops, tumour invasion of the bladder lining should be suspected. Participants should be advised to maintain adequate fluid intake in order to reduce the risk of cystitis.

In cases where the pelvic fields extend inferiorly to include the vulvoperineal region, acute vulvovaginitis may develop. This is managed by applying warm saline soaks, wearing loose clothing and keeping the area dry. Topical steroids and treatment interruption may be necessary also.

4) Dermatologic/Skin (Cutaneous)

Mild irritation and erythema of the skin may develop within the treated areas. More severe reactions, such as desquamation (dry or moist) may develop. The development of acute moist desquamation over the vulvar and perineal skin during the course of external beam radiation is extremely common in areas in which the vulvar/perineal skin is in the treatment fields.

5.2.8. General Radiation Schedule

Day 1	Start whole pelvic radiation therapy
Approximately day 42	Complete whole pelvic radiation therapy
Third week (day 14-21)	Start HDR brachytherapy
Day 46, but no later than day 51	Complete all RT

Patients who have received emergency RT, should be started on the above schedule as soon as possible so the total treatment time does not exceed 56 days.

5.3. Modulated electro-hyperthermia

5.3.1. Dose

mEHT treatments will be administered twice a week with not less than 48 hours between each treatment. This is important in order to reduce the risk of the development of thermo-resistance.

Each treatment will last 55 minutes with a 5 minute preparation time. Each participant will have a total of ten treatments over the five week period during which they receive RT.

mEHT treatments will be administered prior to the administration of RT. RT should be administered as soon after the mEHT treatments as possible and the time between the mEHT treatment and the radiation dose must not exceed thirty minutes.

5.3.2. Administration

The adjustable electrode will be placed over the region of the primary tumour, i.e. over the anterior pelvis, whilst the participant is lying in a supine position on the waterbed containing the static electrode. A sheet must be placed on the waterbed and a sheet or thin paper towel must be placed between the adjustable electrode and the participant to prevent the water bolus on the electrode from touching the skin. Moisture between the participant and the electrodes increases the risk of superficial burns. Participants' perspiration at the site of the applicator must therefore be monitored.

5.3.3. Personnel

The treatments must be administered by a registered nurse, oncology nurse or radiation therapist under the supervision of a medical doctor. For the purpose of this trial, oncology nurses will be asked to administer the mEHT treatment under. Participants must be monitored during mEHT treatments in order to ensure the electrodes are not getting too hot and do not cause burns.

5.3.4. Equipment

The EHY 2000 Plus device with a 30 cm diameter electrode will be used to administer the modulated electro-hyperthermia (mEHT) treatments. The device is manufactured by Oncotherm, a German company and imported into South Africa from the manufacturing office.

The device will be supplied by Oncotherm GmBh and the installation will be done by qualified technicians. Once installed, the device must not be moved or tampered with. Biannual servicing will be done by approved technicians. Under no circumstances are the device modules to be opened or tampered with. The device requires 220 V current and a UPS to protect from lightning and power surges. Any repairs to the device will be done by approved technicians and any faults are to be reported to the manufacturer immediately so that repairs can be done timeously without interrupting the trial.

5.3.5. Quality Control and Documentation

The EHY 2000 Plus will be installed with a web box. The web box allows remote access to the device and the device data. This enables remote monitoring of treatments over internet, as well as remote diagnostics and repairs. All participant data is stored on the web box and will be backed up weekly by the data management team. Treatment duration, interruptions, tumour temperature and energy dose delivered during each treatment for each participant will be entered into the data management system. Hard copies of treatments are also recorded.

5.3.6. Expected Toxicity

The correct administration of treatments ensures that less than 8% of participants develop erythema at the site of the adjustable applicator. Less than 1% of patients report superficial burns at the treatment site. Burn ointment must be kept in stock and nearby in the event that the participants develop burns.

5.3.7. General Modulated electro-hyperthermia Schedule

During Weeks 1 -5 participants will receive 55 minutes of mEHT twice a week, not less than 48 hours apart. The schedules for each week would therefore be one of the following:

- Day 1 & 3/4/5 OR
- Day 2 & 4/5 OR
- Day 3 & 5

6. TREATMENT MODIFICATIONS

In order to ensure the best possible outcome RT and chemotherapy should be administered on time, within the prescribed time frame and in the prescribed doses. However there may be instances when the treatment doses or times cannot be adhered to. This may be as a result of treatment toxicity and dose limiting toxicity. All toxicities will be graded according to the Common Toxicity Criteria (CTCAE v4.0). Principal Investigators must be notified of all Grade 4 CTCAE v4.0 toxicities. Non-medical causes of dose delays may include the participants' inability to get to the hospital on time, non-treatment or disease related illnesses or personal reasons. Participants should therefore be advised that it is in their best interest to adhere to the treatment schedule as closely as possible.

6.1. Cisplatin

All chemotherapy-related toxicities will also be graded according to the Common Toxicity Criteria (CTCAE v4.0).

6.1.1. Cisplatin Modifications

Radiotherapy will not be omitted or delayed for chemotherapy-related toxicities unless the investigator considers that the participant is too sick to be treated.

1) Haematological Adverse Effects

Haematological toxicities are common as a result of treatment with cisplatin. A full blood count with differential must therefore be done on the day of cisplatin administration. Chemotherapy doses will be delayed if the absolute neutrophil count, white blood count or platelets are below the specified values:

**ANC/µL**		**Platelets/µL**
< 1500/mm^3	or	≥ 100/mm^3

*Cisplatin administration after a dose delay can be resumed once the ANC and platelets have increased to 1500/mm^3 and 100/mm^3 respectively.

In the event that treatment must be delayed by more than two weeks the Principal Investigators must be notified and participants must be evaluated. It is not necessary to stop RT during a delay in chemotherapy administration.

2) Gastrointestinal Toxicity

Cisplatin doses will not be modified for diarrhoea as the pelvic radiation will be the most likely cause of diarrhoea in this study. Should pelvic radiation be delayed due to hospitalisation to administer IV fluids due to severe diarrhoea, then cisplatin should not be administered until RT is resumed.

All participants should receive anti-emetics to prevent nausea and vomiting. Anti-emetics should be administered IV prior to each dose of chemotherapy and three days of oral anti-emetics should be supplied for participants. In severe cases of vomiting resulting in hospitalisation and IV fluid and IV anti-emetic administration, cisplatin doses should not be stopped unless RT is delayed. Cisplatin administration should be resumed once the RT is resumed.

3) Nephrotoxicity

Nephrotoxicty will be monitored by serum creatinine tests. If serum creatinine is ≥ 60 on the planned treatment date then the creatinine clearance (CrCl) should be estimated according to the Cockcroft and Gault Equation for women:

CrCl = (140-age in years) (Weight in kg x 0.85) /72 (serum Creatinine)

The following dose modifications and delays are indicated based on the creatinine clearance (a copy of this table will be placed in each participant's study file):

CrCl	Management
60 to 50 mL/min	Hydrate as clinically indicated. Reduce cisplatin dose by 25%.
50 to 40 mL/min	• Hold cisplatin for one week and hydrate as clinically indicated. If repeat CrCl ≥ 50 mL/min after one week, resume with 25% reduction in the cisplatin dose. If CrCl does not recover to ≥ 50 mL/min within one week, permanently discontinue cisplatin.<br>OR<br>• Administer cisplatin at a 50% dose reduction. If CrCl does not recover to ≥ 50 mL/min within one week, permanently discontinue cisplatin.
< 40 mL/min	Permanently discontinue cisplatin.

4) Hypomagnesemia

Hypomagnesemia is not an indication for stopping therapy. Oral or parenteral magnesium supplementation is indicated for serum magnesium levels ≤1.5 mEq/l.

5) Neurological Toxicity

For Grade 1 neurotoxicity, there will be no modification in the cisplatin dose. For participants with ≥ Grade 2 neurotoxicity, hold the cisplatin dose and delay for up to 2 weeks waiting for neurotoxicity to improve to ≤ Grade 1.

- If neurotoxicity recovers to ≤ Grade 1, resume therapy with a dose reduction of cisplatin by 25%.
- If neurotoxicity does not improve to ≤ Grade 1 within 2 weeks, cisplatin will be permanently discontinued.

If ≥ Grade 2 neurotoxicity recurs after 25% dose reduction, cisplatin will be given at a 50% dose reduction upon resolution of neurotoxicity to Grade 0-1.
If ≥ Grade 2 neurotoxicity recurs at 50% dose reduction, or if neurotoxicity does not improve to ≤ Grade 1 within 2 weeks, cisplatin will be permanently discontinued.

6) Ototoxicity

Cisplatin administration should be discontinued permanently for ototoxicty ≥ grade 3 (CTCAE v4.0).

7) Allergic Reactions

Cisplatin administration should be discontinued permanently if ≥ grade 3 anaphylaxis occurs (CTCAE v4.0).

8) Other Grade 3/4 Non-Hematologic Toxicity

Should a participant develop Grade 3 or 4 toxicities (CTCAE v4.0) not already mentioned (excluding nausea, vomiting, anorexia, fatigue, fever without Grade 3/ 4 neutropenia or alopecia), then cisplatin administration should be delayed until the toxicities have resolved to ≤ Grade 1. Subsequent doses of cisplatin should then be reduced.

6.1.2. Completion of Chemotherapy

A total of three doses of cisplatin will be administered. Cisplatin is not to be administered on the day of brachytherapy administration. Cisplatin should not be given after completion of radiation therapy.

6.2. Radiation Therapy

6.2.1. Radiation Dose Modifications

1) Haematological Toxicity

- A white blood count (WBC) of $3000/mm^3$, an absolute neutrophil count (ANC) of $1500/mm^3$ or a platelet count of below $100/mm^3$ necessitate closer monitoring of the participant.
- RT should be stopped if the ANC < $750/mm^3$, WBC < $1000/m^3$, or platlets < $50/mm^3$.
- Febrile neutropoenia requires hospitalisation and intravenous antibiotics. Thrombocytopoenia may require platelet transfusion. In both of these instances the RT will need to be delayed until the symptoms resolve and the haematological parameters described above are met.
- Participants' haemoglobin levels should also be monitored and the levels should be kept above 10gm/dL.

2) Gastrointestinal Toxicity

In the event that participants require hospitalisation in order to manage the gastrointestinal symptoms and administer intravenous fluids, the treatment protocols will need to be temporarily stopped. This must be recorded in the participant's file, along with the cause, and entered into the data management system. Principal investigators must also be notified. The external RT will also be interrupted for grade 3 or 4 gastrointestinal toxicity (CTCAE v4.0). If longer than a two-week break is required, the Principal Investigators should be contacted.

3) Renal/Genitourinary Toxicity

External RT will be delayed for CTCAE v4.0 Grade 3 or 4 bladder toxicity and resumed if the symptoms resolve. In the even that treatment is delayed for longer than two weeks, the Principal Investigators should be notified.

4) Dermatologic/Skin (Cutaneous)

CTCAE v4.0 Grade 1 to Grade 2 dermatologic reactions can be treated with dilute, gentian violet or betnovate cream without interrupting the radiation schedule.

In participants in whom Grade 3 to 4 CTCAE v4.0 dermatologic reactions develop RT should be postponed and the symptoms should be treated appropriately. Management of Grade 3 to 4 CTCAE v.4 reactions includes pain management, warm saline soaks, coating ointments, gentian violet or betnovate and encouraging participants to wearing loose clothing and to keep the area dry. RT should be resumed as soon as the skin reactions have improved to Grade 1 CTCAE v4.0. dermatological manifestations.

Radiotherapy will not be delayed for moist desquamation, unless it is confluent, and if a break is felt to be clinically indicated, the Principal Investigators should be contacted prior to this break.

6.2.2. Dose Delays

One of the study outcomes is the effects of mEHT on the toxicity and tolerability of standard treatments. Participants will therefore not be excluded in cases where dose delays exceed three weeks. However in these instances the Principal Investigators must be notified and an assessment of the cause of the dose delay must be conducted.

Participants must resume treatment as soon as possible in order to ensure the best possible prognostic outcome and all efforts must be made to prevent treatment toxicities and manage symptoms and toxicities in order to avoid extended treatment dose delays.

Chemotherapy should not be administered during a radiation therapy delay.

7. ADVERSE EVENTS MONITORING AND REPORTING

An Adverse Event is defined as any untoward medical occurrence associated with the use of a drug in humans, whether or not considered drug related. Therefore, an AE can be ANY unfavourable and unintended sign (including an abnormal laboratory finding), symptom, or disease temporally associated with the use of a medicinal or investigational product. The adverse event will be attributed as follows: unrelated, unlikely, possible, probable, or definite.

7.1. Classification of Adverse Events

This study utilises the Common Terminology Criteria for Adverse Events (CTCAE) for the reporting and defining of adverse events.

Grade	Description
0	No AE (or within normal limits)
1	Mild; asymptomatic or mild symptoms; clinical or diagnostic observations only; intervention not indicated
2	Moderate; minimal, local, or non-invasive intervention (e.g., packing, cautery) indicated; limiting age-appropriate instrumental activities of daily living (ADL)
3	Severe or medically significant but not immediately life-threatening; hospitalization or prolongation of hospitalization indicated; disabling; limiting self-care ADL.
4	Life-threatening consequences; urgent intervention indicated
5	Death related to AE

7.1.1. Toxicity

Toxicity has been described as an AE that has an attribution of possibly, probably or definitely related to investigational treatment.

7.1.2. Expectedness

An unexpected AE is any AE, the specificity or severity of which is not consistent with documented data regarding the treatment. Additionally the ICH E2A defines an unexpected adverse drug reaction as "an AE, the nature and severity of which is not consistent with the applicable product information (for example, Investigator's Brochure for investigational agent)". All serious adverse events, expected or unexpected, must be reported to the Principal Investigators.

7.1.3. Attribution

The attribution of an AE involves an assessment of the relationship between the AE and the medical intervention. After naming and grading the event, the clinical investigator must assign an attribution to the AE using the following attribution categories:

Relationship	Attribution	Description
Unrelated to investigational agent/intervention	Unrelated	The AE is clearly NOT related to the intervention
	Unlikely	The AE is doubtfully related to the intervention
Related to investigational agent/intervention	Possible	The AE may be related to the intervention
	Probable	The AE is likely related to the intervention
	Definite	The AE is clearly related to the intervention

7.2. Reporting Procedures

All treatment related adverse events must be recorded in the participant files. All AEs with a Grade 2 to 5 must be recorded on the Adverse Events Reporting Form (APPENDIX K). The form is then to be submitted to the data manager and the data will be recorded for analysis at the end of the trial. All serious and/or unexpected events must also be reported to the Principal Investigators.

7.2.1. Routine AE Reporting

All AEs and the attribution and grading of the AEs must be recorded in the participant files. The Data Manager will capture the information for analysis.

7.2.2. Grade 2 and 3 AEs

Principal Investigators must be notified of all Grade 2 and 3 AEs within ten days of the clinician being made aware of the AE.

Any unexpected adverse events associated with RT and any adverse events associated with RT that are a result of user or clinician error must be reported to the necessary governing bodies.

7.2.3. Grade 4 and 5 AEs

Principal Investigators must be notified of all Grade 4 and 5 AEs within 24 hours of the clinician being made aware of the AE.

7.2.4. Deaths

All participant deaths and the cause of death must be documented submitted to the Data Manager within five days.

Deaths that can be attributed to treatment toxicity or AEs must be reported to the Principal Investigators within 48 hours.

7.3. Modulated electro-hyperthermia

All AEs that may be attributed to mEHT treatments must be recorded on the Adverse Events form (see APPENDIX K). The Principal Investigators must be notified of all grade 4 and 5 AEs related to mEHT within 48 hours so that the Principal Investigators can notify the manufacture of the mEHT device, Oncotherm GmbH., of the AEs. At the end of the trial all AEs related to mEHT must be recorded and submitted to the manufacturer.

8. OUTCOME MEASURES

8.1. Primary Outcome Measures

8.1.1. Local Disease Control

For the purpose of this trial, local disease control is considered to be successful if no local recurrence within the pelvic radiation field is noted at the 6 month follow-up PET/CT scan. Persistence of tumour(s) will be considered to be a failure in this measure. Persistence of tumours is diagnosed if there is any clinically documented evidence of recurrence or disease progression within the pelvic field as determined by a PET/CT scan, physical examination and PAP smear.

8.1.2. Progression Free Survival

Progression free survival is defined as the date from protocol registration to date of first documented reappearance (recurrence) or progression of disease or death (cancer related). Progression free survival will be assessed at 6, 12, 18 and 24 months after completion of treatment.

1) Intent To Treat-ITT:

   ITT evaluations will be done in order to ensure that the results are not biased towards the participants who completed the study. This evaluation will include all participants, regardless of whether or not they complete the prescribed treatment protocols.

2) Subset of participants who complete the prescribed chemo-radiotherapy. For this analysis only participants who complete the prescribed RCT will be evaluated.

8.1.3. Two Year Survival

Overall survival is defined as the duration of the participants life from the date of completion of treatment to the death (before two years) or until the two year follow-up with the participant.

8.2. Secondary Outcome Measures

8.2.1. Safety and Tolerability

The evaluation of safety and tolerability will include the safety and tolerability of mEHT and the effects that the addition of mEHT may have on the safety and tolerability of RCT.

1) To evaluate the effect of modulated electro-hyperthermia on treatment toxicity.

   The treatment and control arms will be evaluated in order to determine whether there is a difference in treatment (chemotherapy or RT) safety and tolerability between the two groups and to determine whether the addition of mEHT has any effect on the chemo-radiotherapy toxicity. Participants will also be evaluated in order to determine whether the HIV status of a person has an effect on the toxicity of treatments combined with mEHT.

   The following parameters will be recorded for each participant and used to evaluate the safety and tolerability of chemo-radiotherapy upon completion of the prescribed protocols:

	chemotherapy	RT	Treatment related, non-treatment related or other (explain)	HIV Status
Number of dose delays				
Number of dose reduction				
Number of missed doses				

2) To evaluate the adverse effects associated with modulated electro-hyperthermia.

All adverse effects directly attributed to the mEHT treatments in the study arms must be recorded in the participants' files and recorded in the data management system. At completion of treatments the types and frequency of each adverse effect will be calculated. Participants will also be evaluated in order to determine whether the HIV status of a person has an effect on the adverse effects of mEHT.

8.2.2. Quality of life

Quality of Life will be assessed by means of a QOL survey (see APPENDIX A) which will be conducted before the initiation of treatment, 6 weeks after, and at 3, 6, 9, 12, 18 and 24 months after completion of therapy. Participants' HIV status will also be taken into consideration in order to determine the effects of the addition of mEHT on the QOL of HIV positive participants versus HIV negative participants. Permission has been granted for the academic use of the EuroQOL and EORTC (European Organisation for Research and Treatment of Cancer) Quality of Life questionnaires with a visual analogue scale and specific cervical cancer forms.

8.2.3. Cost per life

The healthcare cost-per-life-per-year will be evaluated by comparing the study and control arms in the HIV positive and HIV negative groups in order to determine whether the addition of mEHT had any long term economic advantages or disadvantages.

The methods used to evaluate the economic implications associated with the addition of mEHT will be:

- QOL
- Visual analogue scale
- Quality Adjusted Life (QUALY) – the life years adjusted based on quality of life. We will use utility valves for this.

Resource sheets (APPENDIX J) will be collected for participants and will document the additional resources used by each participant over and above the standard, protocol driven resources. At the end of the trial the information will be used for an economic analysis of the addition of mEHT to standard treatment protocols. ICER will be used to identify costs incurred during progression.

8.2.4. Effects of treatment on the participant's HIV disease status

As there have, to date, been no studies on the effects of the addition of HT or mEHT on HIV positive patients, and HIV is prevalent in Africa, there is a need to evaluate the safety and effects of HT and mEHT on HIV positive patients in Africa. The effects of the addition of mEHT on the HIV status of patients will be assessed by comparing the following parameters in the HIV positive participants in both arms:

- Full blood count and differential
- CD4 count
- AIDS defining illnesses

8.2.5. Recurrence

A recurrence is defined as increasing clinical, radiological or histological evidence of disease since completion of treatment. Disease recurrence, as well as the recurrence pattern (site of recurrence), will be documented and the results analysed in order to determine the effect of the addition of mEHT to RCT on the recurrence patterns of disease.

8.3. Exploratory Outcome Measures

1) To evaluate thermoradiosensitivity:

   1 tube of 5ml of Heparin Blood will be taken from all four arms of the study before initiating treatment and within one week after completion of treatment. The DNA damage in the lymphocytes will be measured with the micronucleus (MN) assay. The frequency of MN before and after treatment will be a measurement of DNA damage caused by treatment. In this way the DNA damage effects of RCT alone or combined with mEHT on the healthy surrounding tissues can be evaluated.

2) To compare gene expression profiles of lymphocytes and cervical epithelial cells extracted from biopsies before and after mEHT treatment :

   Punch biopsies will be taken of tumours from all four arms of the study before initiating treatment and within 1 week after completion of the treatment. The comparison of the gene expression profiles could lead to establish a predictive method to identify a set of genes related to sensitivity to RCT alone or to the combined treatment with mEHT.

9. CLINICAL AND LABORATORY EVALUATIONS

9.1. Screening Evaluations

9.1.1. Within 14 days prior to initiating protocol therapy

- Physical examination
- Quality of Life survey
- CT scan(without contrast) of pelvis and abdomen
- Chest X-ray
- Ultrasound
- HIV test (ELISA)
- Full blood count with differential
- Urea and Electrolytes (sodium, potassium, chloride, carbon dioxide, magnesium)
- Calculated Creatinine clearance
- Liver function (including AST, ALT and bilirubin)
- Pregnancy test (serum or urine)
- HIV positive participants only
    - CD4+ T-cell count
    - HIV viral load

9.2. Evaluations during Chemo-Radiation Treatment

9.2.1. During Chemo-Radiation

During the five weeks of chemotherapy and radiation treatment participants will require evaluation prior to receiving each dose of cisplatin. ~~This evaluation must be done on the same day as the administration of the cisplatin, i.e. day 1, 21 and 42.~~ The evaluation must include the following:

- Full blood count with differential
- Urea and Electrolytes (sodium, potassium, chloride, carbon dioxide, magnesium)
- Calculated Creatinine clearance

9.2.2. During Brachytherapy

Prior to the administration of each dose of brachytherapy, a physical examination must be conducted on each participant, including vaginal examination, in order to assess the general condition of the participant and the tumour extent.

9.3. Evaluations after Completion of Treatment Protocols

Each evaluation date must be adhered to as closely as possible; however a difference of up to two weeks either before or after will be acceptable.

9.3.1. 3 Months after completion of treatment

- Physical examination
- Quality of Life survey
- Full blood count with differential

- Urea and Electrolytes (sodium, potassium, chloride, carbon dioxide, magnesium)
- Creatinine clearance
- HIV positive participants only
    - CD4+ T-cell count
    - HIV viral load

9.3.2. 6 Months after completion of treatment

- Physical examination
- Quality of Life survey
- Full blood count with differential
- PAP smear
- PET/CT

9.3.3. 9 Months after completion of treatment

- Physical examination
- Quality of Life survey

9.3.4. 12 Months after completion of treatment

- Physical examination
- Quality of Life survey
- PA smear if indicated

9.3.5. 18 Months after completion of treatment

- Physical examination
- Quality of Life survey
- PA smear if indicated

9.3.6. 24 Months after completion of treatment

- Physical examination
- Quality of Life survey
- Full blood count with differential
- PA smear if indicated

9.4. Evaluation of Recurrence

For participants who report a return of symptoms or in whom a recurrence is suspected, the following tests should be done:

- Chest X-ray
- Ultrasound
- PAP Smear
- Quality of Life survey
- Full blood count with differential
- Urea and Electrolytes (sodium, potassium, chloride, carbon dioxide, magnesium)
- Creatinine clearance

- HIV positive participants only, if necessary
    - CD4+ T-cell count
    - HIV viral load

9.5. Off Study Evaluation

Participants who withdraw from the study before completing treatment or before the end of the two year follow up period should be asked to have the following evaluations done as soon as possible:

- Physical examination
- Quality of Life survey
- Tumour measurements and clinical staging
- Chest X-ray
- Full blood count with differential
- Urea and Electrolytes (sodium, potassium, chloride, carbon dioxide, magnesium)
- Creatinine clearance
- 6 month PET/CT
- HIV positive participants only
    - CD4+ T-cell count
    - HIV viral load

10. DATA MANAGEMENT

Once enrolled in the study, participants will be assigned with a study identity number. Participants will be referred to by this number in all documentation and identifying details of participants will not be disclosed.

The participants' medical files will not be removed from the hospital. Copies of the participants' information will be placed in files which will be stored at the data management offices at the Charlotte Maxeke Johannesburg Academic Hospital. The data will be entered into the data management program (RedCap and Excel). Data and information will be backed-up weekly and stored on an offsite hard drive.

10.1. Data Quality

It is the responsibility of the Operations and Data Manager to assure the quality of data for the study. This role extends from protocol development to generation of the final study database.

10.2. Role of Data Manager

The operations and data manager will be responsible for the organisation, filing, storage and back-up of data as well as the recording of data on the Excel document and the backup of the excel document.

11. STATISTICAL ANALYSIS

Analysis will be done by intention to treat. Cox proportional hazards model including each factor (treatment group, HIV status, age, HAART treatment, stage of the disease, dosage of treatments and size of tumour) will be performed to compare the time from randomization to first occurrence of any event (death or recurrence). The hazard ratio and 95% confidence interval will be reported.

Kaplan Meier survival curves will be plotted at 6 months, 1 year and at 2 years (for endpoints death and survival). Logrank statistics will be used to compare both treatment arms. Overall typeI error is considered at 5%.

11.1. Outcome variables

11.1.1. Primary

1) Local disease control at 6 months assessed by PET/CT scan, PAP smear and a physical examination.
2) Progression free survival at 6, 12 ,18 and 24 months in all four groups
   a) ITT – everyone
   b) Only those completing study
3) Overall survival at 2 years
4) Cause of death
   a) Cancer
   b) Treatment related
   c) HIV related
   d) Other

11.1.2. Secondary

1) Adverse events associated with mEHT
2) Effects of mEHT on the toxicity and tolerability of chemotherapy and RT evaluated by:
   a) Adverse events
      - Chemotherapy
      - RT
   b) Dose delays and causes of dose delays
      - Chemotherapy
      - RT
   c) Dose reductions and causes
      - Chemotherapy
      - RT
   d) Missed treatments and causes
      - Chemotherapy
      - RT
   e) Acute and late complications
      - Chemotherapy
      - RT
3) Quality of Life assessment at start, 6 weeks, 3, 6, 9, 12, 18 and 24 months
4) Economic evaluation

5) To evaluate the effect, if any, of mEHT treatments on the HIV disease status of HIV positive participants.
   - CD4 count
   - HIV viral load
   - Concurrent AIDS-defining conditions

6) To describe cervical cancer recurrence patterns in all groups
   - Loco-regional
   - Distant recurrences

11.2. Tumour characteristics

- Stage Tumour size (> 2 cm)
- Pelvic nodal status
- Histology (squamous or adeno-squamous)

11.3. Participant characteristics

- Age (>70)
- Performance status (ECOG 1-2)
- Disease stage (IIB – III)
- Full blood count plus differential
- Urea and Electrolytes (sodium, potassium, chloride, carbon dioxide, magnesium)
- Creatinine clearance
- HIV status and CD 4 count, viral load and AIDS defining illnesses

11.4. Sample Size Considerations

We plan to recruit 236 participants (we expect a drop out of approximately 15%) from the Charlotte Maxeke Hospital over forty eight months and will have a minimum of two years of follow-up. We anticipate at least 50% of recruited participants will be Stage III and around 30% will be HIV positive. *(updated to 72 months, n=271, drop out/screening failure n=22.5%)*

11.5. Site(s) of Recurrence

The site(s) of first disease recurrence will be classified as: pelvic-only, extrapelvic- only, pelvic-and-extra-pelvic or no recurrence, and will be tabulated by treatment group.

11.6. Toxicity Analysis

The toxicity data will be tabulated by treatment. The scale will be the Common Toxicity Criteria grading system (i.e., 0-5) but combining Grades 0, 1 and 2. Five toxicity categories (CTCAE v4.0) will be tested: gastrointestinal (nausea, vomiting diarrhoea), genito-urinary (acute toxicity manifested by cystitis, and acute vaginitis), and dermatologic (inguinal area, vulva, perineum, acute rash/desquamation, generalized macular, vesicular eruptions, and ulcerative dermatitis), blood/bone marrow and neurologic toxicities. Fisher's exact test will be employed to assess observed frequency differences for (incidence of maximum grade) specific adverse events by treatment arm.

11.7. Statistical Analysis Plan

The Kaplan-Meier method will be used to describe the progression-free survival (PFS). We will generate KM curves for STAGE IIB advanced (i.e. distal half of parametria infiltrated) and IIIB participants. The binomial proportion and its 95% confidence interval will be used to estimate the objective response rate for the treatment.

The Cochran-Mantel-Haenszel chi-square test will be used to compare HAART use, compliance, node positivity (including enlarged pelvic nodes,) and other known risks that affect survival with respect to objective response rate across all strata.

Frequency tables will be used to summarize the occurrences of adverse events by severity grade. Frequencies of specific adverse events will be tabulated.

Toxicity assessment: All toxicities will be reported on standard case report forms at visits and graded by NCI CTCAE v4.0. There will be standard dose reduction schedules. All dose delays, dose reductions, and missing doses will be tracked via CRFs during therapy. These will be tabulated and reported as rates.

12. EXPLORATORY RESEARCH

Molecular Thermoradiosensitivity Research

MEHT should ideally potentiate the DNA damaging effect of RCT in cancer cells and reduce it in normal cells. Because DNA damage repair can depend on temperature, mEHT can modulate the sensitivity to RCT of normal and cancer cells. Chromosomal damage induced by ionising radiation and heat (thermoradiosensitivity) can be tested through a variety of cytogenetic assays. One of these tests is the Micronucleus (MN) assay. Micronuclei (MN) are small nuclei that form in the cytoplasm when chromosomes or chromosome fragments are not incorporated into the daughter nuclei subsequent to cell division. In the MN assay, cell division is stimulated by the addition of the mitogen, phytohaemagglutanin (PHA), and cytokinesis is blocked by adding cytochalasin B. Micronuclei are counted in cells that have undergone a single nuclear division i.e. a binucleated cell. Due to its ease, efficiency and reliability, the MN assay is commonly used in biological dosimetry and chromosomal radiosensitivity studies. Recent automation of MN scoring with the Metafer platform (Metasystems) has further improved this method by allowing it to become especially rapid and reproducible, due to the withdrawal of subjective MN scoring by technical staff (Willems et al., 2010).
To investigate the molecular markers for thermoradiosensitivity gene expression profiles of thermoradiosensitive and thermoradioresistent tumours will be compared. The identification of genotypes correlated with sensitivity to RCT alone or combined with mEHT will be of great benefit to the practice of radiotherapy, and could eventually result in individualised treatment schedules. This set of genes could be also useful in separating patients with and without recurrence following mEHT.

Blood samples and biopsies will be collected from each participant before initiating treatment and within 7 days of completion of the treatment regimes.

The exploratory translational research will be conducted by Dr Ans Baeyens and PhD student Olivia Herd, Radiobiology lab, department of Radiation Sciences, University of the Witwatersrand.

Participants will be made aware of extra translational research part of the project and will be asked permission with an extra informed consent and questionnaire (see APPENDIX H) to use the samples for this type of research.

13. ETHICAL AND REGULATORY CONSIDERATIONS

13.1. Informed Consent (see APPENDIX G and H)

In South African medical law the informed consent means that the participant is given sufficient knowledge and appreciation of the situation presented in order to be able to make a knowledgeable and objective decision on the topic. It ensures the participant's right to free choice and self-determination. The informed consent gives the participant an understanding of the purpose, procedures, nature, scope, consequences, risks, dangers, complications, prognosis and alternatives of participating in the trial. It is the doctor's duty to disclose the information to the participant in a manner in which the participant understands and can base decisions on. [16]

The informed consent form provides the participants with the necessary information on the benefits and disadvantages of participation in the trial as well as the process, protocols and implications involved in participating in the trial. The informed consent form is based on the guidelines as outlined by the Health Professions Council of South Africa (2007). [17] No participant will be allowed to enter the trial without reading, understanding and signing an informed consent. All participants must have the informed consent verbally explained in order to ensure that they understand the contents of the form. Should the form not be written in the participant's language, the form must be translated verbally and explained to the participant in a language which the participant understands. Each participant will be given an informed consent form consisting of two parts: Information sheet and a Consent form. The voluntarily signed consent form will be returned to the investigators and the information sheet will be given to the participant. The participants will be asked to allow the researchers' permission to access their medical records for research purposes.

13.2. Changes to the Protocol

Any change or addition to this protocol requires a written protocol amendment that must be approved by the Principal Investigators before implementation. Amendments significantly affecting the safety of subjects, the scope of the investigation or the scientific quality of the study, require additional approval by the Human Research Ethics Committee. In this case a copy of the written approval must obtained in writing before the changes can be implemented.

13.3. Women and Minorities

No minors will be enrolled in this trial and the rights and respect of all the women enrolled in the trial will be ensured at all times.

13.4. Subject Confidentiality

In order to ensure strict confidentiality of all participants at all times, all documentations relating to participants, including files, forms and reports, and all specimens will only contain the participants three digit trial ID number. The participants name and identifying information will not appear on any of the documents or records kept and used in this trial. The details of the participants will not be released to anyone not involved in the trial without written consent from the participant.

13.5. Study Discontinuation

This study may be discontinued at any time by the Principal Investigators, the Department of Health and the ethics committee should they at any point feel the participants are at risk or the trial has been compromised.

APPENDIX A QUALITY OF LIFE QUESTIONNAIRE

Permission was granted from the relevant bodies to use the following Quality of Life instruments for academic research purposes:

EuroQol Group:

EQ-5D-5L
https://euroqol.org/

European Organisation for Research and Treatment of Cancer:

EORTC QLQ 30 version 3
EORTC QLQ CX34 (cervix specific)

https://qol.eortc.org/

APPENDIX B ECOG

ECOG PERFORMANCE STATUS*	
**Grade**	**ECOG**
0	Fully active, able to carry on all pre-disease performance without restriction
1	Restricted in physically strenuous activity but ambulatory and able to carry out work of a light or sedentary nature, e.g., light house work, office work
2	Ambulatory and capable of all selfcare but unable to carry out any work activities. Up and about more than 50% of waking hours
3	Capable of only limited selfcare, confined to bed or chair more than 50% of waking hours
4	Completely disabled. Cannot carry on any selfcare. Totally confined to bed or chair
5	Dead

* As published in Am. J. Clin. Oncol.: *Oken, M.M., Creech, R.H., Tormey, D.C., Horton, J., Davis, T.E., McFadden, E.T., Carbone, P.P.: Toxicity And Response Criteria Of The Eastern Cooperative Oncology Group. Am J Clin Oncol 5:649-655, 1982.*

Eastern Cooperative Oncology Group, Robert Comis M.D., Group Chair.

APPENDIX C BODY MASS INDEX TABLE

Table 1: The International Classification of adult underweight, overweight and obesity according to BMI

**Classification**	**BMI(kg/m^2)**	
	**Principal cut-off points**	**Additional cut-off points**
**Underweight**	**<18.50**	**<18.50**
Severe thinness	<16.00	<16.00
Moderate thinness	16.00 - 16.99	16.00 - 16.99
Mild thinness	17.00 - 18.49	17.00 - 18.49
**Normal range**	**18.50 - 24.99**	**18.50 - 22.99**
		**23.00 - 24.99**
**Overweight**	**≥25.00**	**≥25.00**
Pre-obese	25.00 - 29.99	25.00 - 27.49
		27.50 - 29.99
**Obese**	**≥30.00**	**≥30.00**
Obese class I	30.00 - 34.99	30.00 - 32.49
		32.50 - 34.99
Obese class II	35.00 - 39.99	35.00 - 37.49
		37.50 - 39.99
Obese class III	≥40.00	≥40.00

Source: Adapted from WHO, 1995, WHO, 2000 and WHO 2004.

APPENDIX D ELIGIBILITY CHECKLIST AND ENROLMENT FORM

ENROLMENT FORM

STUDY ID ___________ Date of enrolment: ___________

First names ____
Last name ____
ID number ____
GT Number ____
DXT Number ____
Date of birth ____
Home address ____
Postal address ____
Work Address ____
Contact number Cell ____________________________________
Home ____________________________________
Work ____________________________________

Patient age: _____________ Patient Gender: _____________
Home Language: ___________ Second Language: ___________

Carcinoma of the cervix Squamous OR adeno-squamous
Stage ___________________________________

Next of Kin Name ____________________________________
Relationship ____________________________________
Contact number____________________________________
Next of Kin Name ____________________________________
Relationship ____________________________________
Cell ____________________________________
Home number ____________________________________
Work number ____________________________________

Monthly income? - None
- less than R500
- between R500 and R1000
- between R1000 and R2000
- between R2000 and R5000
- more than R5000
- unknown

Highest grade completed? Primary school
High school
Tertiary school

Cigarette use – number of cigarettes per day: ____________
Number of years: ____________

Patient name: ___________________________________ Study no: ______________________________

GT: ____ Date:________________________________

Has the patient had a recent blood transfusion? ☐Yes ☐ No
If yes, please state when, and why: ____

Was Emergency Radiation Therapy required to Stop Bleeding? ☐Yes☐ No
* In the event that emergency radiation is required to stop the bleeding of tumours prior to commencing the treatment protocols, radiation treatment should be started within two weeks after the administration of emergency radiation so the total treatment time does not exceed 56 days.

Age of patient (must be between 18 and 70years of age) __________
Has the patient been treated for cervical carcinoma before? ☐Yes ☐No
Has the patient been treated for any other invasive cancer within the last 24 months? ☐Yes ☐No
If your previous answer is yes, please explain:____

Have the following investigations been done:

Physical assessment ☐ Yes ☐ No

Chest x-ray ☐ Yes ☐ No
Suspicious nodes (N) ☐ Yes ☐ No
Abdominal Ultrasound (Y) ☐ Yes ☐ No
Hydronephrosis (N) ☐ Yes ☐ No RHS or LHS

ECOG score	: ___________	Height	:_________________
BMI	: ___________	Weight	:_________________
HIV	: ___________	Stage	:_________________

Are the following tests within the specified ranges?

- WBC 4.00-10.00 Value: ________
- Haemoglobin >10 g/dL; Value: ________
- Platelet count >150/mm^3; Value: ________
- ANC >3000/mm^3 Value: ________
- Lymphocytes 1.00-4.00 Value: ________
- ALT 7-35 U/L Value: ________
- AST 13-35 U/L Value: ________
- Total Bilirubin > 2 X umol/L Value: ________
  Unless related to antiretroviral use then the direct bilirubin must be < 2 X ULN.
- Urea 2.1-7.1 Value: ________
- Creatinine 49-96 Value: ________
- Creatinine clearance>60 mL/min Value: ________

Have you included a copy of the relevant histology and cytology reports? ☐ Yes ☐ No
Are there para-aortic lymph node visible on CT? ☐ Yes ☐ No
Does the patient understand the informed consent? ☐ Yes ☐ No
Is the patient willing and able to sign the informed consent? ☐ Yes ☐ No
Have you confirmed via appropriate tests the patient is not pregnant? ☐ Yes ☐ No
Is the patient breast-feeding? ☐ Yes ☐ No
Has the patient had a hysterectomy? ☐ Yes ☐ No
Is the patient able to bear children? ☐ Yes ☐ No
Is the patient prepared to take measures to prevent pregnancy? ☐ Yes ☐ No
Do you know the patient's HIV status? ☐ Yes ☐ No
Have you included the patient's latest HIV test results? ☐ Yes ☐ No
Does the patient have a life expectancy of longer than 12 months? ☐ Yes ☐ No
If the patient is HIV positive:

a) Is she on ARTs? ☐ Yes ☐ No

b) What is the CD4 count? ____________

c) Is she receiving treatment at an HIV clinic? ☐ Yes ☐ No

d) If yes, what treatment is she receiving?____

e) Have you referred her to an HIV clinic? ☐ Yes ☐ No

f) Any life-threatening AIDS defining illness (excluding cervical carcinoma) ☐ Yes ☐ No

If your previous answer is yes, please explain:_______________________________________

____

Does the patient have any contra-indications to the standard treatment protocols? ☐ Yes ☐ No

Does the patient have any contraindications to Oncothermia treatments? ☐ Yes ☐ No

e) Pace makers and other implanted devices which rely on current/charges ☐ Yes ☐ No

f) Large metal implants, such as hip replacements. ☐ Yes ☐ No

g) Inability to feel temperature in the region. ☐ Yes ☐ No

h) Inability to express or vocalise discomfort or heat at the treatment site. ☐ Yes ☐ No

Does the patient have any life-threatening, severe, uncontrolled infections? ☐ Yes ☐ No

Does the patient appear to be able to adhere to treatment protocols? ☐ Yes ☐ No

PET Measurements WIDTH:__________ HEIGHT: __________ SAGITTAL: __________

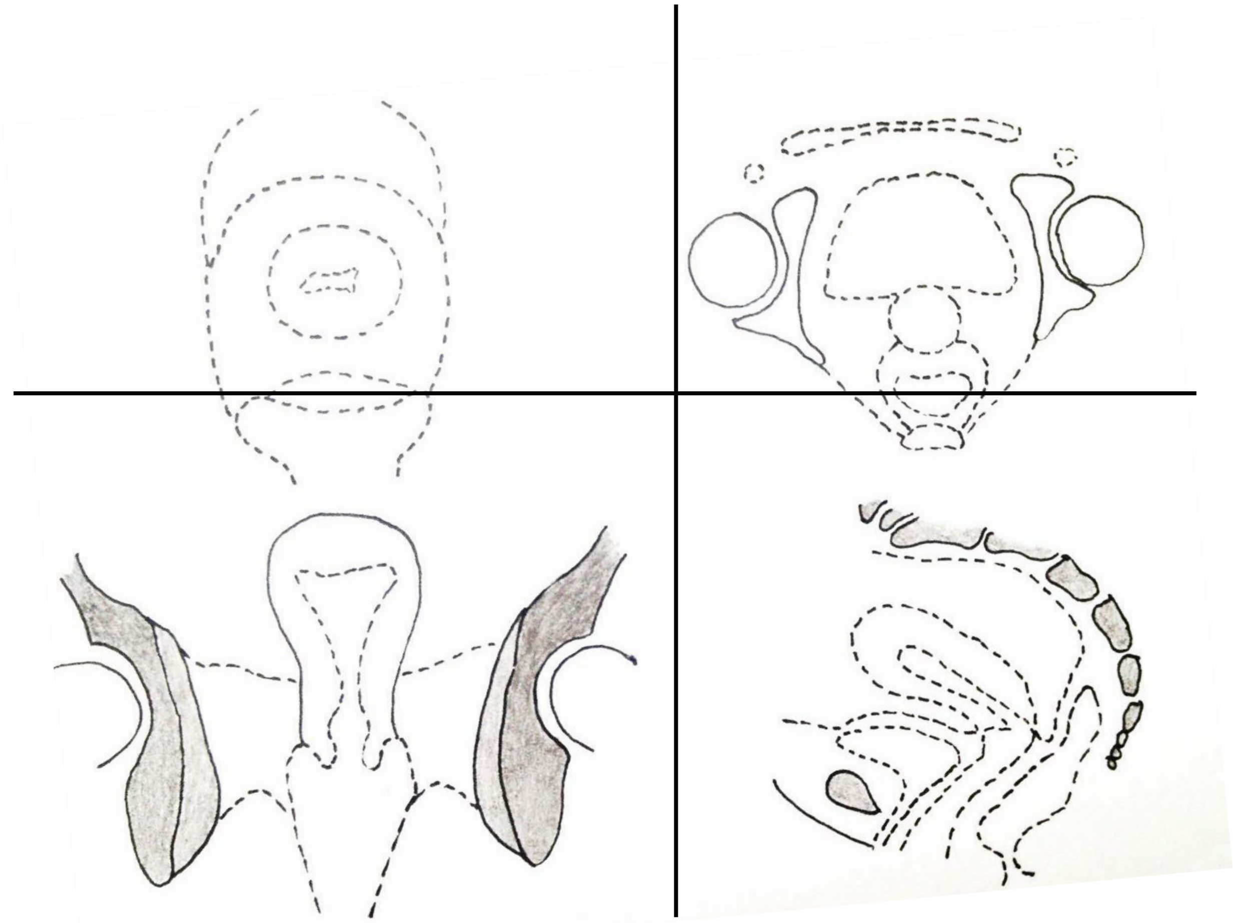

Name of Physician: ___________________________ Signature of Physician: ____________________

APPENDIX E INFORMATION AND CONSENT FORM

Study title: A randomised trial investigating the benefits of the addition of Modulated electro-hyperthermia to chemo-radiation for cervical cancer in HIV positive and negative women in South Africa
Principal Investigators: Jeffrey Kotzen, Carrie Minnaar
Institution: University of the Witwatersrand and Charlotte Maxeke Academic Hospital

Information Sheet:

"***A randomised trial investigating the benefits of the addition of Modulated electro-hyperthermia to chemo-radiation for cervical cancer in HIV positive and negative women in South Africa***".

Dear Prospective Participant

Dr. Jeffrey Kotzen and Carrie Minnaar are conducting a clinical trial at the Charlotte Maxeke Johannesburg Academic Hospital. We are working together with the University of the Witwatersrand Medical School and a small group of doctors and staff members at the radiation oncology department at the Charlotte Maxeke Johannesburg Academic Hospital. Oncology is the treatment of cancer. In this clinical trial we are investigating a type of therapy called hyperthermia. Hyperthermia means hot. In oncology, hyperthermia therapy means to heat up tumours. We will be using a medical device (machine) to heat up tumours. We want to see if this helps the treatments for cervical cancer to work better.

The title of our clinical trial is:

"***A randomised trial investigating the benefits of the addition of Modulated electro-hyperthermia to chemo-radiation for cervical cancer in HIV positive and negative women in South Africa***".

A clinical trial is a method of testing new types of treatments or therapies. Clinical trials involve participants who voluntarily choose to take part in the clinical trial. This means that it is entirely your choice whether or not to participate in the clinical trial. We are inviting you to take part in our clinical trial. You have been asked because you have advanced cervical cancer and this is the cancer that we will be testing the effects of hyperthermia on.

Before you make your decision, you need to know about the treatments being offered, the risks and the benefits, what will be expected of you and what is expected of us. This document contains all the information that you need to make a decision. You may have more questions after reading this information sheet. Your study doctor and the study investigators will be available to answer all of your questions before you make a decision. Our contact details are at the end of this information sheet and you may contact us with any further questions that you may have relating to this study. It is important that you have all the information you need to make a decision. It is also important that you are kept up to date with any new information that affects you or the clinical trial. It is our responsibility to make sure that you are kept informed of any new information that we get during the trial.

If you decide to take part in the trial, you will be asked to sign a consent form. You will also be given a copy of this information sheet to take home and refer to at a later stage.

Why are we doing this clinical trial?

The purpose of this trial is to see if the normal treatment for advanced cervical cancer can be improved if the tumours are heated before they are treated. If the treatment is improved, we want to see if this helps women with advanced cervical cancer to live longer. This is very important, especially

in South Africa, where we have thousands of women treated for cervical cancer every year. The results of this trial will help doctors in the future to decide whether or not heating the tumours is helpful.

How does the trial work?

We will be inviting 236 women between the ages of 18 and 70 who have been diagnosed with advanced cervical cancer to participate in the trial. Each woman who participates will receive the standard treatment for advanced cervical cancer. Standard treatment means the normal treatment that all women with advanced cervical cancer have access to at the Charlotte Maxeke Academic Hospital. The normal treatment involves three (3) doses of chemotherapy, five weeks of radiation therapy and three doses of brachytherapy. This is the standard treatment for advanced cervical cancer and it is given because it is currently the best way that we know of to treat advanced cervical cancer. These treatments are explained in more detail in the next few pages. It is important to know that these treatments are given to all women with advanced cervical cancer, even if they are not a part of this trial.

Half of the women in the trial will receive only the standard, or normal, treatments. This means that they will not receive anything else that is not standard or normal for women with advanced cervical cancer. Any risks and benefits for these women will be the same as the risks and benefits for women being treated for advanced cervical cancer who are not involved in the clinical trial. This group of women will be known as the control group.

The other half of the women will receive the standard treatments and will also receive the hyperthermia treatments. This group will be known as the treatment group.

Each woman who agrees to participate in the trial will be randomly assigned to either the control group or the treatment group. This means that neither the women nor the study doctor(s) will know which group you will be entered into at the beginning of the trial. This is important because it means that the study doctor(s) cannot control or decide which group a woman will be assigned to and can therefore not affect the results of the study. Only after each woman has been randomly assigned to a group, will the woman and the study doctors will be told which group the woman has been assigned to.

If you agree to be part of the trial, you will be given the standard treatment either with or without hyperthermia. The treatments last about six (6) weeks. After the treatment has been finished, we will ask you to return to us for regular check-ups so that we can see how well the treatment worked and how well you are feeling. We will be checking on you for two years after you have finished your treatment. At the end of the trial we will compare the health of the group of women who did not receive hyperthermia with the group of women who did receive hyperthermia so that we can see which group of women showed the most benefits.

Does your HIV status affect your involvement in the trial?

If you want to participate in the trial we will need to know what your HIV status is. This means that, unless you have already had a recent HIV test, we will need to do an HIV test on you before we start the treatment.

HIV causes damage to the parts of your body that help you to stay healthy. Some of the medicines that are used to treat cancer also cause damage to the parts of your body that help you to stay healthy. This is why it is important to know your HIV status before you are given the medicines to treat your cancer.

If you are HIV positive, some blood will be used to test the viral load and the CD4 count in your body. These two tests will tell us if you are healthy enough to have the medicine for your cancer. If the tests tell us you are not healthy enough for medicine then we will refer you to an HIV clinic where you will be given medicines called antiretroviral treatments, or ARVs, to help you to fight the virus. Once you are on these medicines and your health improves, then you can have the medicine to treat the cancer. You may be asked by the doctors at the HIV clinic to visit them regularly for tests and for medication. In order to be a part of the trial we need your permission to contact the HIV clinic for copies of any tests that they do on you and to find out what medicines they are giving you. This will help us to make sure you stay healthy and to understand the best way to treat you and other HIV positive patients with cervical cancer.

The results of your HIV tests will also be used in the trial and will be made available to the study doctors and people working on the trial. Your HIV test results will be kept strictly confidential, just like the rest of your results.

What treatments will be given?

If you are randomly assigned to the treatment group then you will receive all of the standard treatments (chemotherapy, radiation therapy, brachytherapy) and examinations plus hyperthermia treatments. If you are randomly assigned to the control group then you will receive the standard treatments (chemotherapy, radiation therapy, brachytherapy) without hyperthermia.

Regardless of which group you are assigned to, you will also still receive any other treatments and medications that you may need, such as medicine to stop you from feeling sick and medicine to help manage any pain that you might have.

Chemotherapy:

Chemotherapy is a type of medication that is used to treat cancer. The type of chemotherapy used to treat advanced cervical cancer at the Charlotte Maxeke Academic Hospital is called Cisplatin. Cisplatin is given to you through a needle that is put into a vein. Cisplatin is poisonous to cancer cells and helps the radiation therapy to destroy the cancer cells.

Radiation therapy:

Radiation is an invisible energy that is made by a large machine. The radiation machine can control where the energy goes. It is similar to a microwave, but much bigger and more complicated. Just like a microwave makes energy which cooks food, radiation machines make energy which helps to destroy tumours. You will receive radiation therapy for a few minutes every Monday to Friday for five weeks. The radiation energy will be put into the area of your body where the tumour is.

Brachytherapy:

Brachytherapy is another type of radiation but this type is given inside your body, and not outside your body. This means that it is put inside your vagina. You will be given brachytherapy three times.

Hyperthermia:

If you are assigned to receive hyperthermia, the hyperthermia treatments will be given to you twice a week during the five weeks that you receive radiation therapy. Each treatment lasts only one (1) hour and will be given before your daily dose of radiation therapy. This means that if you are in the hyperthermia group, then on the days that you receive hyperthermia treatments you will be asked to come to the hospital two (2) hours before your radiation appointment. Having hyperthermia does not hurt. You will be asked to lie on a bed and a round device that feels like a pillow full of water will be placed over the area of your body where your cancer is. You will be asked to lie still for an hour and during this time you will feel that your skin will get warm. It is a very comfortable treatment and it should not hurt you at all. If it gets too hot, the doctor or nurse giving you the treatment will turn the temperature down.

What will you need to do to participate in the trial?

You have been given this information sheet because your doctor, after diagnosing and examining you, feels that you are suitable for the clinical trial. If you decide to participate in the trial you will be asked to sign a form that gives us your written permission to include you in the trial. The tests and examinations that will be done are the same tests and examinations that everybody with your kind of cancer has done.

There will however be some things that we will ask you to do that are not part of the standard treatment. These are:

You will be asked to give regular feedback and answer questions on how you are feeling so that we can monitor your health. This will be done by completing questionnaires called Quality of Life forms. You will be asked to complete these forms before your treatment is started, straight after you have finished your treatment, every three months for the first year after your treatment and every six months during the second year after your treatment. These forms can be completed at the hospital with your study doctor or over the phone if you are unable to come to the hospital on that day.
You will be asked to have a CT scan before you start treatment and six (6) months after your treatment has been completed. CT scans are special machines which are able to take pictures of the inside of your body. The pictures that we get from your scans will show us whether your tumours are getting smaller, getting bigger or staying the same size.

Before you receive treatment, the following will be required:

- Physical examination – during the examination your study doctor will check your health and make sure that you are able to participate in the trial.
- Quality of Life form
- Special machines will be used to help the doctors have a look at the cancer inside your body. These machines are called CT scanners, X-ray machines and an ultrasound. Each of these machines gives us a special picture of what is happening inside you and helps us to plan your

treatment. These machines do not cause any pain and they are all very safe. You cannot have pictures done with these machines if you are pregnant though as it could cause the baby harm.

- About 20 ml of your blood samples will be taken and tested to make sure that you are healthy enough to participate in the study. Taking this amount of blood is safe and will not harm you at all.
- Some of the blood that is taken will also be used to test you for HIV.
- You will also be asked to do a pregnancy test. The standard treatment for cervical cancer is very dangerous for the babies of pregnant women. It is important to make sure that you are not pregnant before you start the treatment.

During your treatment:

Everyone in the trial will receive the same treatment as all other woman with advanced cervical cancer like yours. During your treatment your doctor will take samples of your blood at least another three (3) times to make sure that the chemotherapy dose that the doctors are giving you is safe for you. The doctors may also examine you to make sure that the radiation therapy and brachytherapy is not causing any serious side effects.

After your treatment is complete:

You will be examined regularly and will have regular blood tests to monitor your health during the two years after you have finished your treatment. You will be asked to come in and see your study doctor every three months during the first year and every six months during the second year after your treatment. Your final appointment will be two years after the treatment has been finished. These appointments are important and will allow your study doctor to make sure that your treatment is working and that you are healthy. You will also be asked to have another CT scan and another PAP smear six (6) months after you have completed treatment and will be asked to have an x-ray one year and two years after you have finished your treatment.

If you are HIV positive then your doctor may also ask you to have another test three months after completing your treatment to check that the HIV is still controlled.

Your Information and Confidentiality

All of the information relating to your health will be recorded in your medical files which are kept at the hospital. The study doctors will have access to your files in order to monitor your health. The study doctors will make copies of your blood tests, quality of life questionnaires, imaging (CT, x-ray and ultrasound) results and other medical information. However once the study doctor has made copies of your reports, your name will be taken off all the reports and replaced with your reference number. In order to participate in the trial you will be asked to give us permission to use your medical records for the clinical trial. We need these records in order to compare your health to everybody else's health on the trial and to see which group of women has the best results after two years.

The study doctor(s) will do everything possible to make sure that your information is protected and kept private. Each woman in the trial will be given a number. Only the study doctors will have the list that shows which person has which number. The file that is made for you for the trial will only have a number on it and your name and personal details will be taken off all the information in the study file. This means that no one other than the study doctors treating you will know who you are.

It is important to know that every effort will be made to make sure that your results are kept confidential. However there may be times when regulatory bodies require the study doctors to release the names of the participants. Regulatory bodies are groups of people who control and monitor all the clinical trials in South Africa in order to make sure that the study doctors and researchers are looking after the participants properly. They may also want to make sure that the study investigators are being truthful about the results of the trial. It is important that all trials are monitored so that all participants are looked after and the results of the trials are accurate. If a regulatory body requests your information from the study doctors and investigators, the regulatory body will also make every effort to maintain your confidentiality. Sometimes the study doctors will be required to release your information to authorities such as law enforcement agencies. If this happens it will be done in accordance with the law and everyone involved will handle your information with discretion and will work hard to make sure that your information remains private.

You are allowed to talk about your treatment to anyone that you want to. You are not required to keep your treatment or your participation in the trial a secret.

If you participate in the trial, what is your responsibility?

It is your responsibility to ensure that you attend all of your treatment appointments and receive all the treatments prescribed to you. It is important that you understand that the standard treatments are given with your best interest in mind and they are your best chance of managing your cancer. It is therefore important that you make every effort possible to ensure that you attend each treatment appointment in order to make sure that your health is managed properly. This would be the same whether you are participating in the trial or not. It is your responsibility to make sure that you are available to receive your treatment. If you skip too many appointments or are unable to attend your appointments then we may discontinue your participation in the trial. This is because we need results from people who have followed the treatments properly so that at the end of the trial we can make the correct decision about which treatment was best. However if you are unable to attend your treatments you will not in any way be discriminated against. If your participation in the trial is discontinued you will still have access to treatment just like everybody else who is not participating in the trial.

What if you fall pregnant?

The treatments for cervical cancer are very dangerous to the babies of pregnant women and can be harmful to the children of breast feeding mothers. It is therefore your responsibility to make sure that every effort is made to prevent yourself from falling pregnant while you are being treated for cervical cancer. It is also important that you do not breast feed while you are receiving the treatment. If you suspect that you are pregnant you must tell your study doctor straight away so that the study doctors can make sure that your baby is not harmed by the treatments.

Your options for preventing pregnancy include the following:

- Birth control pills
- Female condoms
- Diaphragms
- Ensuring that your male partner wears male condoms
- Birth control injections
- Intrauterine device (IUD)
- Not having sex

What will it cost to participate in the clinical trial?

You will not be asked to pay for any of the treatments that you receive during the trial. The costs for you will only be the costs for transport and travelling to the hospital to receive your standard treatments. You will be reimbursed for the transport costs for your follow up visits every three months during the first year and every six months during the second year, as these are not part of the standard treatments.

What are the risks of taking part in the clinical trial?

The risks with the type of hyperthermia that we are using are very small. In eight (8) out of one hundred (100) people the skin, where the machine touches your body, turns red for up to half an hour after the treatment. One (1) out of a hundred (100) people will have mild burns where the machine touches the body. This risk is very small and every attempt will be made by your study doctor to minimise this risk. If you do get a burn we will make sure that you burns are treated correctly.

The risks associated with the standard treatments are the same risks that any other person may have when being treated for advanced cervical cancer. These risks include:

Risks associated with Cisplatin:

Common:	Decreased immune function, infections, mouth sores, anaemia, tiredness, weight loss, decreased appetite, nausea, vomiting, diarrhoea, constipation, kidney problems, hair loss, tingling or pain in your fingers and toes, changes in your taste sensations, mouth sores, ringing sound in your ears and a skin rash.
Uncommon:	Allergic reaction, severe infections, bruising and bleeding, changes in your ability to see, some cancers, deafness, heart problems, liver problems,

Risks associated with Radiation Therapy:

Diarrhoea, Constipation, vaginal bleeding, difficulty urinating, skin rashes and pain in the area being treated, vaginal pain, painful sex, decreased sexual function, loss of your pubic hair.

Non-study medications

There is also a risk that you may have side effects to some of the medications that are given to you during the study but are not for the treatment of your caner. Examples of these medications are medicines which stop you from vomiting, medicines for pain, medicines which stop diarrhoea etc. Mixing certain medications can also be dangerous. It is important that you tell your nurse or doctor all of the medicines that you are taking so that we can make sure that mixing the medicines with the chemotherapy does not make you sick.

While we have listed all the risks that we can think of above, there may be some risks which we are not aware of or do not know about. This clinical trial will help us to identify any risks that we may not yet know of.

What are the benefits to participating in the study?

There are several benefits that you may experience if you participate in the study. However these benefits cannot be guaranteed. If you participate and you are assigned to the treatment group you may experience the benefits of the hyperthermia. Previous clinical trials suggest that having hyperthermia with your normal treatment will improve your overall health and help you to live longer. These benefits cannot be guaranteed.

If you are not assigned to the hyperthermia group, you will still benefit from the close monitoring and care that you will receive whilst participating in the trial.

Can the study doctors stop your participation in the trial?

The study doctors will always have your best interests in mind and may therefore decide to stop your treatment, without your permission, if they feel that the treatment is harming you, is not benefiting you or if you become pregnant. The study doctors may need to stop your participation in the trial if the trial is stopped or cancelled or if you are not able to attend all of your treatment and follow up appointments.

What if you decide not to participate in the trial?

If, after reading through this information sheet, you decide not to participate in the trial, your treatment and healthcare will not in any way be affected. You will still receive the same standard treatment that everyone else with your disease receives. Healthcare is your right and a decision not to participate in this trial will not in any way affect your right to healthcare. It is also your right to choose whether or not to participate in the clinical trial. Your participation in entirely optional.

You also have the right to change your mind once you have been entered into the trial. You can decide to stop participating in the trial at any time for any reason. If you decide to stop participating in the trial you will still receive standard treatment for your cancer. You will still have access to all of the treatments that every other woman with advanced cervical cancer who is not participating in the trial has access to.

What are your other options for treatment?

If you choose not to participate in this clinical trial you can still receive all the normal treatments and care that women with advanced cervical cancer have access to. You also have the right to choose not to have any treatment for your cancer. However these decisions must be discussed with your doctors so that you are sure that you make the right decision for your health.

Thank you for your time and please feel free to contact us if you have any further questions.

Best regards,

Dr. Jeffrey Kotzen (011) 481 2137
Study doctor and Principal Investigator

Carrie Minnaar (072) 1234 292
Co-investigator study co-ordinator

Consent Form:

"***A randomised trial investigating the benefits of the addition of Modulated electro-hyperthermia to chemo-radiation for cervical cancer in HIV positive and negative women in South Africa***".

Study participant number: Date of Birth:

Contact no:

I have been given a copy of the information sheet.
I have read the information sheet it or it has been read to me and explained to me.
All of my questions have been answered to my satisfaction.
I understand what is involved in participating in the clinical trial and I understand all the risks and benefits associated with participating in the clinical trial.
I agree to participate in the clinical trial and to make my medical information relevant to the trial available to the investigators.
I have also agreed that the study doctors may access my medical records and medical files.
If I am HIV positive I have agreed to give the study doctors permission to contact the HIV clinic for my test results.

____
Print Name of Participant

____ ____________
Signature of Participant Date

____
Print Name of Witness

____ ____________
Signature of Witness Date

In the opinion of the Investigator, the participant, is capable of complying with this protocol?
☐ Yes ☐ No

____
Print Name of Investigator Obtaining Consent

____
Signature of Investigator Obtaining Consent Date

APPENDIX F EXPLORATORY INFORMED CONSENT FORMS

Study title: Analysis of thermo- and radiosensitivity in South African cervical cancer patients.
Investigators: Dr A Baeyens, Olivia Herd and Carrie Minnaar
Institution: WITS University and NRF-iThemba LABS

Contact numbers:	Dr A Baeyens: 011 481 21 60;	072 919 88 72
	Olivia Herd: 082 778 4929	Carrie Minnaar 072 123 4292

Good Day,

We are Dr. A. Baeyens, Carrie Minnaar and Olivia Herd from the Radiobiology research unit, Department of Radiation Sciences and NRF- iThemba LABS based at Charlotte Maxeke Johannesburg Academic Hospital, Orange block, 4th floor. We are investigating the radiosensitivity and thermoradiosensitivity of cervical cancer patients. Thermoradiosensitivity refers to how well the radiation alone or in combination with heat works on your tumour and normal organs. This is assessed by the increased susceptibility of cells, tissues or organs to the harmful effect of ionizing radiation. Ionizing radiation is used in radiotherapy to treat cancer.

Information on individual radiosensitivity helps to monitor the radiotherapy treatments.

We invite you to consider participating in a research study titled 'Analysis of thermo- and radiosensitivity in South African cervical cancer patients'. Your participation in this study is entirely voluntary. If you decide to take part in this study, you will be asked to sign this document to confirm that you understand the study. You will be given a copy to keep.

Should you decide not to participate in the study or if you agree and then change your mind, there will be no implications for you and the best treatment available for you will still be given.

If you agree to participate, we kindly ask you to donate 5ml of blood twice – once before your treatment and once during the third week of your treatment. This is not a lot of blood; it is only 1 teaspoon and will not harm you. We will use your blood sample to test the sensitivity to radiation and oncothermia.

We also kindly ask you if we take a sample of your tumour using a procedure known as a biopsy before you start your treatment and again during the third week of your radiation treatment. A biopsy may cause you discomfort.

These samples will be used to investigate if there is a link between the thermo- and radiosensitivity seen in blood and in cervical cells. Both your blood sample and tissue samples will be used to unravel the underlying mechanism of radiosensitivity. We also ask permission to view your medical files if we need to obtain any further medical information that may be relevant to our study.

There is no direct benefit to you. But your participation in this study will contribute to the development of greater knowledge of radiosensitivity and may help to further develop the radiotherapy treatments of cervical cancer patients.

The research is completely confidential, which means that your name will not be recorded on any of our laboratory information. The consent forms will be locked away and only accessible by the researchers. We will require some personal details from you (your age, language, monthly income, see questionnaire attached) and we also want to know if you are a smoker or if you have any other major illness, as this can have an influence on our tests.

You are free to ask any questions about this study and discuss any worries you may have with the research staff.

Thank you very much for your time

Regards,

Dr A. Baeyens, Olivia Herd and Carrie Minnaar

Study title: Analysis of thermo- and radiosensitivity in South African cervical cancer patients.
Investigators: Dr A Baeyens, Olivia Herd and Carrie Minnaar
Institution: WITS University and NRF-iThemba LABS
Contact numbers: Dr A Baeyens: 011 481 21 60;072 919 88 72 Olivia Herd: 082 778 4929

Study participant number: Date of Birth:

Contact no:

INFORMED CONSENT:

I hereby confirm that I have been informed about the nature, conduct, benefits of the study on radiosensitivity of cervical cancer patients

I have also received, read and understood the above written information regarding this study

I have no further questions and declare myself prepared to participate in the study.

PARTICIPANT:

Name (Print):

Signature and date:

STUDY STAFF CONDUCTING CONSENT DISCUSSION:

Name (Print):

Signature and date:

WITNESS (IF APPLICABLE):

Name (Print):

Signature and date:

APPENDIX G FIGO STAGING CRITERIA

Carcinoma of the Cervix

IA1	Confined to the cervix, diagnosed only by microscopy with invasion of < 3 mm in depth and lateral spread < 7 mm
IA2	Confined to the cervix, diagnosed with microscopy with invasion of > 3 mm and < 5 mm with lateral spread < 7mm
IB1	Clinically visible lesion or greater than A2, < 4 cm in greatest dimension
IB2	Clinically visible lesion, > 4 cm in greatest dimension
IIA1	Involvement of the upper two-thirds of the vagina, without parametrial invasion, < 4 cm in greatest dimension
IIA2	> 4 cm in greatest dimension
IIB	With parametrial involvement
IIIA/B	Unchanged
IVA/B	Unchanged

Pecorelli S. (2009) **Revised FIGO staging for carcinoma of the vulva, cervix, and endometrium**. *International Journal of Gynaecology and Obstetrics* Vol. 105, No. 2, pp:103-4

APPENDIX H RESOURCE SHEET

This sheet is to document all treatments and resources utilised which are outside of the scope of these protocols and are not considered to be a part of standard practice.

Date: __________________ Patient number: __________________

Medication Pain medication type, quantity and reason:____________________________________
____
Antibiotics – type, quantity and reason:____
____
Treatment for skin reactions type, quantity and reason: _________________________
____
Anti-emetics – type and quantity:____
____
Anti-diarrhoeal – type and quantity: ____
____
Iron supplements and quantity: ____
____
Other:____
____

Blood transfusion (quantity):____

Hospital admission: Reason, duration and details:
____
____

Surgery or procedures: (include reason, duration, sedation/anaesthetic)
____
____

Other: ____
____
____

Signature : ___________________________

APPENDIX I ADVERSE EVENTS

Date	Details	Grade	Expected or unexpected?	Treatment related (Definitely, very likely, maybe?)	If treatment related, which treatment?	Management

REFERENCES

[1] Fiorentini G. and Szasz A. (2006) **Hyperthermia today: Electric energy, a new opportunity in cancer treatment** *Journal of Cancer Research Therapy* Vol. 2, No. 2, pp: 41-46

[2] Van der Zee J. (2002) **Heating the patient: a promising approach?** *Annals Oncology* Vol. 13, No. 8, pp: 1173-1184

[3] Van der Zee J., Gonzales Gonzales D., Van Rhoon G.C., *et al.* (2000) **Dutch Deep Hyperthermia Group Comparison of radiotherapy alone with radio therapy plus hyperthermia in locally advanced pelvic tumours: a prospective randomised multicentre trial** *Lancet* Vol. 355, pp: 1119-1125

[4] Franckena M., Stalpers L.J.A., Koper P.C.M., *et al.* (2008) **Long-term improvement in treatment outcome after radiotherapy and hyperthermia in loco-regionally advanced cervix cancer: an update of the Dutch Deep Hyperthermia Trial** *International Journal of Radiation, Oncology, Biology and Physics* Vol. 70, No. 4, pp. 1176–1182

[5] Van der Zee J. and Gonzalez G.D. (2002) **The Dutch Deep Hyperthermia Trial: results in cervical cancer** *International Journal of Hyperthermia* Vol. 18, No. 1, pp: 1-12

[6] Harima Y., Nagata K., Harima K., Ostapenko V.V., Tanaka Y. and Sawada S., (2001) A **randomised clinical trial of radiation therapy versus thermoradiotherapy in stage IIIB cervical carcinoma** *International Journal of Hyperthermia* Vol. 17, No. 2, pp: 97-105 Kansai Medical University, Japan

[7] Franckena M., Stalpers L.J.A., Koper P.C.M., *et al.* (2008) **Long-term improvement in treatment outcome after radiotherapy and hyperthermia in loco-regionally advanced cervix cancer: an update of the Dutch Deep Hyperthermia Trial** *International Journal of Radiation, Oncology, Biology and Physics* Vol. 70, No. 4, pp. 1176–1182

[8] Franckena M., Lutgens L.C., Koper P.C., *et al.* (2009) **Radiotherapy and hyperthermia for treatment of primary locally advanced cervix cancer: results in 378 patients** *International Journal of Radiation, Oncolgy, Biology and Phsyics* Vol. 73, No. 1, pp: 242-250

[9] Lutgens L., van der Zee J., Pijls-Johannesma M., et al. (2010) **Combined use of hyperthermia and radiation therapy for treating locally advanced cervical carcinoma** *Cochrane Database of Systemic Reviews* Vol. 20, No. 1, CD006377, Maastro Clinic, Maastricht, Netherlands, Available online from: http://www.ncbi.nlm.nih.gov/pubmed/20091593

[10] Franckena M., SE Wit R., ansink A.C., *et al*. (2007) **Weekly systemic cisplatin plus locoregional hyperthermia: an effective treatment for patients with recurrent cervical carcinoma in a previously irradiated area international** *Journal of Hyperthermia* vol. 23, no. 5, pp: 443-450 Shahrekord University of Medical Sciences, Iran available online from: http://www.ncbi.nlm.nih.gov/pubmed/17701535

[11] Westermann A.M., Jones E.L., Baar-Christian S., van der Steen-Banasik E.M., Koper P., Mella O., Uitterhoeve A.L.J., de Wit R., van der Velden J., Burger C., van der Wilt C.L., Fahl O., Prosnitz L.R., van der Zee J. (2005) **First Results of Triple-Modality Treatment Combining Radiotherapy, Chemotherapy, and Hyperthermia for the Treatment of Patients with Stage IIB, III, and IVA Cervical Carcinoma** *American Cancer Society* DOI 10.1002/cnr.21138, Netherlands, U.S.A., Norway

[12] Tsuda H., Tanaka M., Manabe T., Ikeda H., Negoro S., Ishiko O. and Yamamoto K. (2003) **Phase I study of combined radiation, hyperthermia and intra-arterial carboplatin for local recurrence of cervical cancer** *Annals of Oncology* Vol. 14, pp: 298–303

[13] Chen H., Wang Y., Fan J., Si L., Lou W. and Li M. (2005) **The Impact of Hyperthermotherapy plus Radiochemotherapy for Cervical Carcinoma on Long-term Survival and Toxicity** *Journal of US-China Medical Science* Vol. 2, No.4, (Serial No.5)

[14] Chen H.W., Jun-Jie F., Wei L. (1997) **A randomized trial of hyperthermo-radiochemotherapy for uterine cervix cancer** *Chinese Journal of Clinical Oncology* Vol. 249, pp: 249-251

[15] Sreenivasa G., Hildebrandt B., Kummel S., Jungnickel K., Cho C.C., Tilly W., Bohmer D., Budach V., Felix R. and Wust P. (2006) **Radiochemotherapy combined with regional pelvic hyperthermia induces high response and resectability rates in patients with nonresectable cervical cancer figo iib** *International Journal of Radiation, Oncology, Biology and Physics*, Vol. 66, No. 4, pp: 1159-1167

[16] Carstens P.A. (n.d.) **Informed consent in South Africa medical law with reference to legislative developments**, *Published by the South African Medico-Legal Society* Available online from: http://new.samls.co.za/node/354 (Accessed October 2011)

[17] Health Professions Council of South Africa (2007) **Seeking the patients informed consent: Ethical considerations** Available online from: http://www.hpcsa.co.za/downloads/conduct_ethics/rules/seeking_patients_informed_consent_ethical_consideration.pdf (Accessed October 2011)
